# Defensive behavior is linked to altered surface chemistry following infection in a termite society

**DOI:** 10.1038/s41598-023-42947-9

**Published:** 2023-11-23

**Authors:** M. Alejandra Esparza-Mora, Tilottama Mazumdar, Shixiong Jiang, Renate Radek, Julian N. Thiem, Linshan Feng, Vesta Petrašiūnaitė, Ronald Banasiak, Marek Golian, Melanie Gleske, Christophe Lucas, Andreas Springer, Jan Buellesbach, Dino P. McMahon

**Affiliations:** 1 Institute of Biology, Freie Universität Berlin, Königin-Luise-Straße 1-3, 14195 Berlin, Germany; 2 Department for Materials and Environment, BAM Federal Institute for Materials Research and Testing, Unter den Eichen 87, 12205 Berlin, Germany; 3 Institute for Evolution and Biodiversity, University of Münster, Hüfferstraße 1, 48149 Münster, Germany; 4 Institut de Recherche sur la Biologie de l’Insecte (UMR7261), CNRS–University of Tours, Tours, France; 5 Core Facility BioSupraMol, Department of Biology, Chemistry and Pharmacy, Freie Universität Berlin, Takustraße 3, 14195 Berlin, Germany

**Keywords:** Ecology, Evolution, Zoology

## Abstract

The care-kill response determines whether a sick individual will be treated or eliminated from an insect society, but little is known about the physiological underpinnings of this process. We exploited the stepwise infection dynamics of an entomopathogenic fungus in a termite to explore how care-kill transitions occur, and identify the chemical cues behind these shifts. We found collective responses towards pathogen-injected individuals to vary according to severity and timing of pathogen challenge, with elimination, via cannibalism, occurring sooner in response to a severe active infection. However, injection with inactivated fungal blastospores also resulted in increased albeit delayed cannibalism, even though it did not universally cause host death. This indicates that the decision to eliminate an individual is triggered before pathogen viability or terminal disease status has been established. We then compared the surface chemistry of differently challenged individuals, finding increased amounts of long-chained methyl-branched alkanes with similar branching patterns in individuals injected with both dead and viable fungal blastospores, with the latter showing the largest increase. This coincided with the highest amounts of observed cannibalism as well as signs of severe moribundity. Our study provides new mechanistic insight into the emergent collective behaviors involved in the disease defense of a termite society.

## Introduction

The evolutionary and ecological success of social insects is widely appreciated. Although sociality offers many benefits, there are also associated costs^1^. On the one hand, cooperation facilitates division of labor, enabling the optimization of tasks such as brood care, foraging, nest building, information sharing and defense^2–4^. On the other hand, densely populated and closely related individuals inside a confined nest environment are at risk of disease transmission^5,6^. The risk of repeated pathogen exposures have shaped the evolution of defense mechanisms across insect societies^7^. Social insect individuals are not only able to fight infections through their own individual immune systems^8–10^, but also via social immune defenses^2,3,11^. Social immunity can be viewed as a distributed organ, similar to the immune system of metazoan animals^12^ that builds on two principal adaptive pillars—behavior and physiology—that together act to prevent the entrance, establishment, and spread of pathogens in a colony^2,4,6,9,13,14^.

Social insect groups, such as Hymenoptera (social bees, wasps, and ants) and Isoptera (termites) contain multiple lineages of species with advanced social living, often referred to as superorganisms^15^. Studies in these social groups have demonstrated the range of collective disease defenses that can be deployed against infected individuals^3,16–20^. Infected individuals can be groomed, which in combination with the use of antimicrobial secretions, serves to rescue infected individuals by reducing the likelihood of internal infection. In contrast, once an internal infection is successfully established, it can pose a threat to the colony and the individual may need to be eliminated^3,6,17,21^. Such radically different responses towards infected individuals have been described in terms of a care-kill dichotomy^6,21,22^. Allogrooming is one of the first lines of defense against external pathogens, while elimination typically occurs at a later stage^17,19,23–25^. Both strategies are thought to reduce the risk of disease outbreak and enhance colony survival^3,6^, but the latter imposes an additional cost to the colony through the loss of workforce members, depending on worker age as well as colony size and maturity.

Collective disease defenses are known to vary within and between species and across different levels of social organization^3,12,26,27^. Detection of pathogens and the regulation of the collective response depends on the ability of social organisms to accurately communicate disease-related information through behavioral or chemical cues^6,28^. Chemical communication is thought to play a central role in social immunity^6^. For instance, in the ant *Lasius neglectus*, destructive disinfection behaviors are employed, whereby ants initially detect fungus-infected pupae via sickness cues emitted by the pupae, followed by elimination^19^. Signalling and chemical communication in social insects can be mediated by cuticular hydrocarbons (CHCs)^29^. CHCs are a predominant class of chemical compounds coating the cuticle of terrestrial insects and have a key role in inter- and intraspecific communication^30,31^, potentially also serving to discriminate between healthy and sick nestmates within a colony^29^. CHC profiles can be altered by activation of the immune system^29,32–34^ and altered CHC profiles have been implicated as signals to eliminate infected individuals in ants and honeybees^19,29,32,35^ in a manner analogous to body cells signalling their status to the immune system^36^.

Termites also employ several collective mechanisms to prevent disease outbreaks, largely using two complementary behaviors: allogrooming and cannibalism, reflecting the care-kill strategy mentioned above. The switch in the response depends on the progression of the infection, with cannibalism occurring when infected individuals display signs of overt sickness, such as slow movement^17^. Application of antimicrobial secretions, in addition to behavioral responses, can effectively remove external pathogens from the cuticle of infected individuals and inhibit pathogen growth during the early stage of infection^23,37–44^. However, when a pathogen successfully initiates a potentially lethal internal infection^45^, such weak or moribund but not yet dead individuals are typically cannibalized^46,47^. Cannibalism is thought to be ancestral to all termites, and may have evolved as a prophylactic mechanism against disease from an initial role in nitrogen recycling^48^. Still, the basis of the switch between social immune behaviors in termites remains largely unknown, and it is unclear what signals or chemical cues trigger elimination behaviors or to what extent they are linked with factors associated with moribundity^17^.

To address these gaps in our knowledge, we investigated how termites detect and respond to internal infection upon injection of either viable or dead blastospores of a generalist fungal pathogen. Fungal blastospores, which represent a within-host infectious form, play an important role in pathogenesis during disease development and their experimental injection can elicit a robust immune response^49–52^. Blastospores are a vegetative cell state that can quickly multiply and colonize the insect body^53–55^. Furthermore, blastospores reflect an intermediate stage in the infection process*,* which begins with conidia adhesion and germination on the insect cuticle, followed by cuticular penetration via appressoria to colonize the host’s body. Within the host, the fungus proliferates as single-celled blastospores or hyphal bodies in the hemolymph, ultimately killing the infected insect^45,53,56,57^. During infection, virulence factors are synthesized, such as destruxins (cyclodepsipeptidic mycotoxins) and even host miRNAs can be manipulated^58^ to inactivate the host and assist in avoidance of immune clearance^49,52,54,59,60^.

Here, we combine blastospore injection and behavioral experiments with chemical analyses of termite CHC profiles and fungal destruxins to expand our understanding of termite social immunity. Our rationale is that to better study the care-kill switch, it is useful to separate direct external pathogens from indirect host-mediated cues, allowing one to better investigate how termites detect and respond to an internal pathogen challenge. We explore how defensive behaviors are triggered by experimentally varying internal pathogen challenges, and correlate these with alterations to termite surface chemistry. Our study sheds new light on a key collective defensive mechanism of a termite society.

## Results

### Blastospores germinate quickly and synthesize destruxins

**Figure 1:**
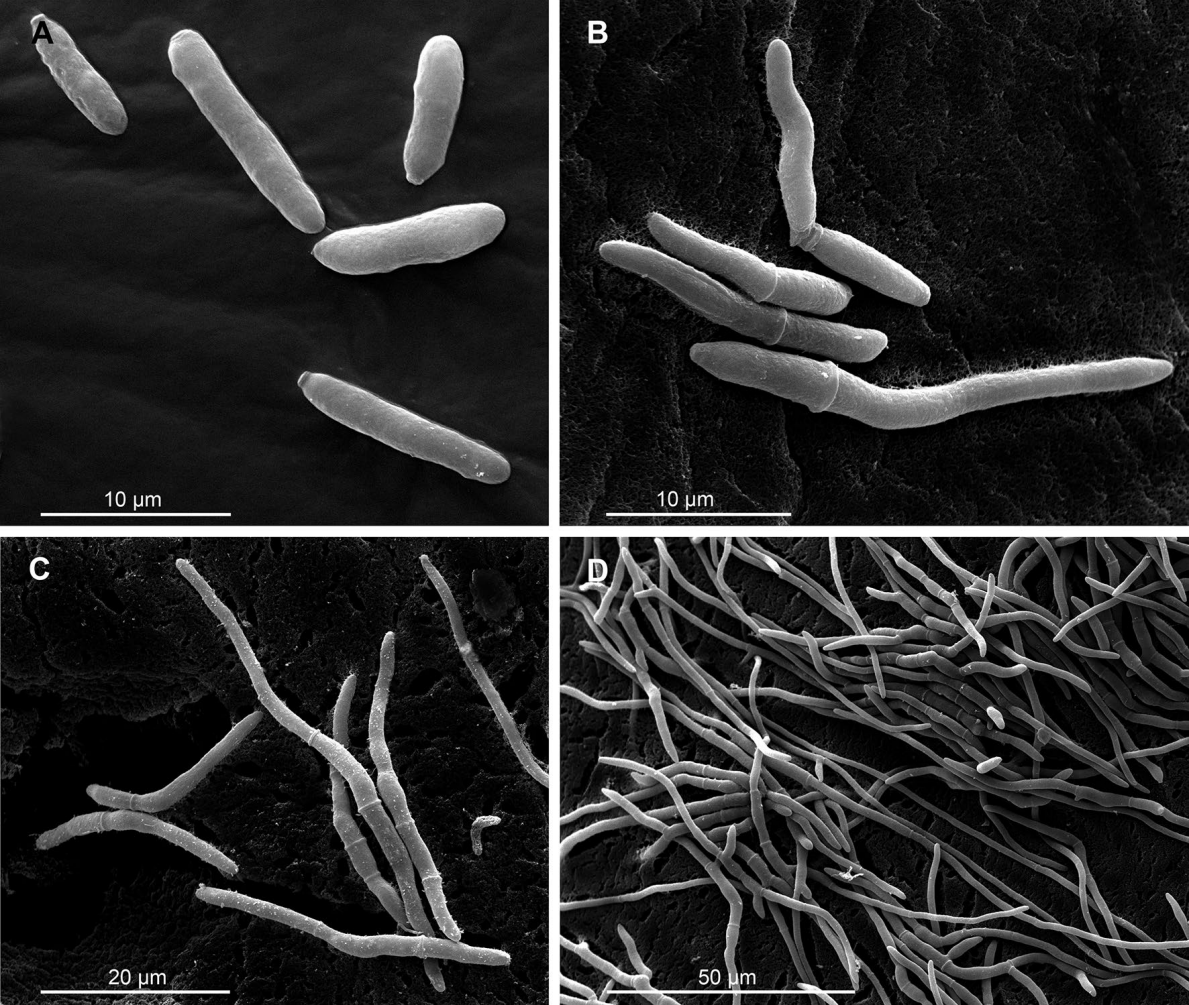
Germination process of *M. robertsii* 1490 blastospores. (**A**–**D**) Scanning electron micrograph (SEM) images of blastospore germination at different time points post-inoculation in vitro (PDA medium). At 2 h blastospores are not yet germinated (**A**), while at 4 h, germination of blastospores can already be observed (**B**), which continues to an advanced stage via germ tube elongation at 8 h (**C**). By 12 h, elongation of the fungal hypha has continued, and mycelial networks are now visible (**D**).

We applied *Metarhizium robertsii* blastospores via injection to exclude the presence of external pathogen cues (cuticular conidiospore exposure) from internal host activity (infection initiation). Prior to injection experiments, we examined rates of blastospore germination. We used a scanning electron microscope (SEM) to quantify germination (Fig. 1A–D) on potato dextrose agar (PDA) plates. Germination was observed as early as 4 h after inoculation, as evidenced by the formation of germ tubes (Fig. 1B, Supplementary Fig. S1). Generally, the earliest time reported in the literature is 2.7 h in the phylogenetically divergent fungus *Isaria fumosorosea*^61^. At 8 h, germination and elongation of germ tubes continued and by 12 h a thick mass of elongated hyphae, forming the mycelium (Fig. 1C, D) could be observed. This rate of in vitro blastospore germination is faster than either in vitro or in vivo (on cuticle) conidia germination^17,45^ (Supplementary Figs. S1, S2). In addition to viable blastospores, we employed dead blastospores as an injection treatment. We confirmed the efficiency of inactivation following heat-treatment (autoclaving) by the absence of germination after plating. The presence of 5 major classes of destruxins: (Destruxin A, B, C, D, E and Ed) were also investigated in the filtered media Czapek-Dox (CD) modified medium of cultured viable *M. robertsii* blastospores (Supplementary Fig. S3). Comparisons of the UHPLC-MS/MS data with the elution order and the MS/MS spectra published in the literature^62,63^ verified the the identities of the respective destruxins found in the chromatograms, confirming their presence. By contrast, no destruxins were detected in the filtrate derived from heat-inactivated (dead) blastospores (Supplementary Fig. S4), indicating that destruxins were destroyed by autoclaving.

### Both viable and dead blastospore injection causes termite death

Before embarking on behavioral experiments in our study species, the eastern subterranean termite *Reticulitermes flavipes*, we first measured survival of individual termites when kept in isolation, in order to measure treatment lethality in the absence of group dynamics. Ringer-injection had no negative effect on termite survival, whereas viable blastospores caused 100% mortality by 2.5 days post injection, and dead blastospores resulted in a plateau at 40% mortality by 5 days post injection (Supplementary Fig. S5) that remained unchanged for 10 days, at which point observations were terminated.

### Termites slightly increase allogrooming following exposure to injected individuals

In the first behavioral observation experiment, we compared the responses of experimental colonies to termites injected with Ringer’s solution or blastospores (viable or dead). Injected focal termites were isolated for 2, 8, 12 and 15 h prior to introduction into experimental colonies. Behavioral patterns were then recorded in response to Ringer’s solution (Ringer), viable (Blastospores+) or dead blastospore (Blastospores−) injected focal termites at different stages post-challenge. Observed behaviors in response to blastospore injected focal termites consisted of low levels of grooming, high amounts of cannibalism and no observations of burial (Supplementary Fig. S6), as well as other behavioral states unrelated to social immunity. Behavioral patterns in the control treatments were characterized mostly by other states, low levels of grooming and one incident of cannibalism in the 15 h incubation time.

**Figure 2:**
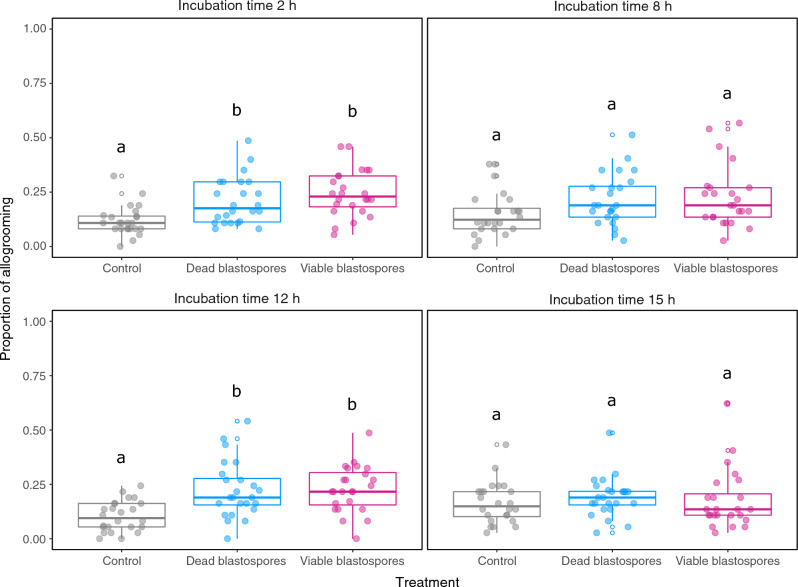
Allogrooming as a proportion of total observation states across treatments in the blastospore experiment. The proportion of allogrooming states differs significantly between treatments for incubation time points 2 and 12 h. Significant post hoc comparisons are indicated by different letters, derived from separate GLMMs from each time point. Note that post hoc comparisons do not apply between incubation time points. Lower and upper hinges correspond to first and third quartiles. Upper and lower whiskers extend to the largest/smallest value if no greater/smaller than 1.5 times the inter-quartile rage from the hinge, respectively.

**Figure 3:**
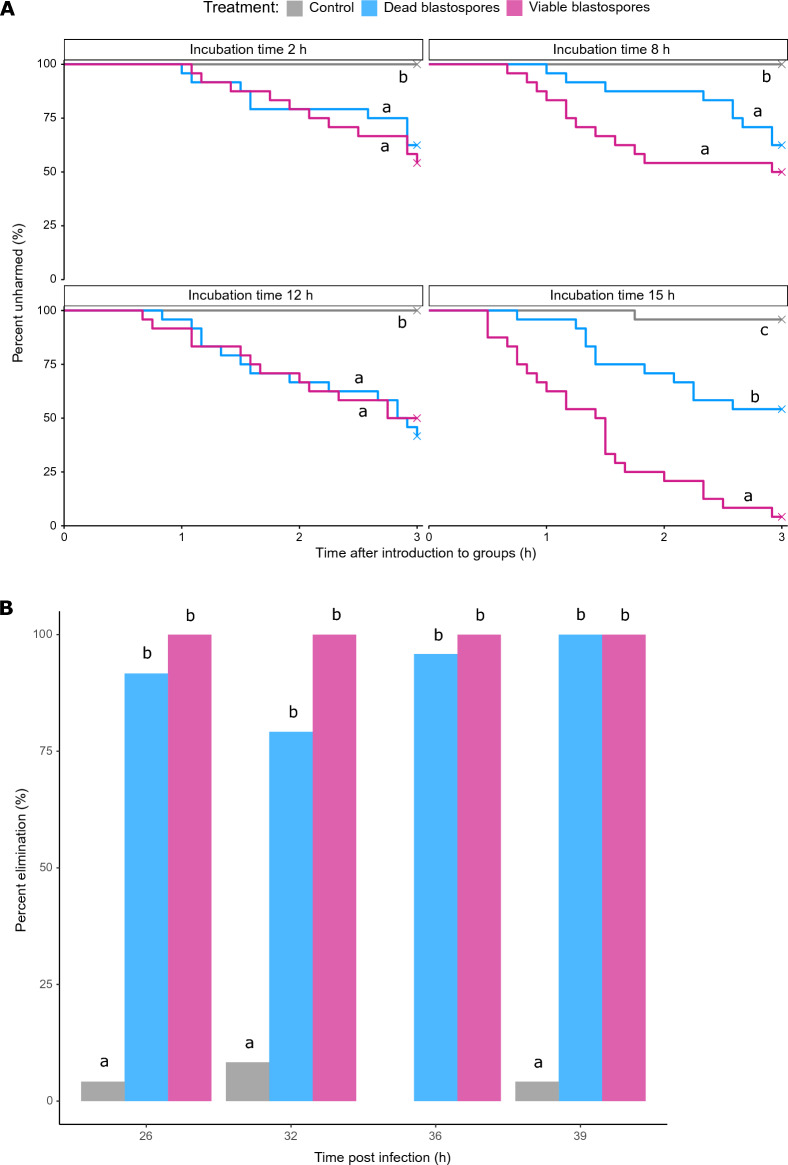
Survival of focal termites following introduction into experimental colonies. (**A**) Percentage of focal termites that remain unharmed (due to cannibalism) after different incubation times (2, 8, 12, 15 h). Cannibalism states were recorded over a three-hour observation period following introduction into experimental colonies. Crosses indicate the presence of right-censored data (i.e. focal termites that were not dead or harmed during the observation period). Significant differences between treatments derived from survival models are indicated by different letters. Note that post hoc comparisons do not apply between incubation time points. (**B**) Percentage of focal termites that had been eliminated at 24 h following their introduction into experimental colonies, corresponding to 26, 32, 36 and 39 h post-injection for incubation time points 2, 8, 12 and 15 h, respectively. Note that at equivalent time points for dead blastospore-injected termites kept in isolation (Supplementary Fig. S5), mortality was lower than 20%. Treatments: Grey = Ringer (control), Blue = dead blastospores, Pink = viable blastospores.

The overall proportion of allogrooming in both viable and dead blastospore treatments were significantly higher than control treatments, except at 8 h and 15 h (Supplementary Table S1) (2 h, Blastospores− vs Ringer *z* = 3.293 *P* = 0.0030, Blastospores+ vs Ringer  *z* = 4.448 *P* < 0.0001; 12 h, Blastospores− vs Ringer *z* = 4.324 *P* < 0.0001, Blastospores+ vs Ringer *z* = 4.515 *P* < 0.0001). The low proportion of allogrooming observed at the later 15 h time point coincides with a high proportion of cannibalism (Figs. 2, 3A, Supplementary Table S1, Supplementary Fig. S7).

In a follow-up experiment, we compared injection of dead blastospores with injection of an equivalent solution filtered to remove blastospore debris (Filtrate). The overall proportion of allogrooming was at its highest at 12 h post-challenge and decreased over time for the two fungal treatments (Blastospores− and Filtrate) while remaining unchanged for the control group, with no significant differences detected between any of the time points. The overall proportion of allogrooming in both dead blastospore and filtrate treatments was significantly higher over control treatments at two incubation time points (12 h, Blastospores− vs Ringer *z* = 7.636 *P* < 0.0001, Filtrate vs Ringer *z* = 5.349 *P* < 0.0001; 15 h, Blastospores− vs Ringer *z* = 6.626 *P* < 0.0001, Filtrate vs Ringer *z* = 4.634 *P* < 0.0001) (Supplementary Fig. S8). Allogrooming was significantly higher in dead blastospore versus filtrate treatment at 12 h but at no other time point (12 h, Blastospores− vs Filtrate *z* = − 2.510 *P* = 0.0363). A progressively lower level of grooming corresponded with an increasingly higher proportion of cannibalism by 15 and 18 h time points, resulting in elimination by 21 h post infection for a high number of termites (Supplementary Table S3 and Supplementary Figs. S9, S10).

### Cannibalism does not completely depend on termite moribundity

In the first experiment, the highest amounts of cannibalism were observed at 15 h post injection in the viable blastospores treatment, with cannibalism beginning shortly after introduction of the focal termite, and completely replacing grooming before the end of the observation period (Supplementary Fig. S6). Interestingly, we observed that nestmates cannibalized dead blastospore-injected focal individuals as well (Fig. 3A, Supplementary Table S2). However, cannibalism occurred sooner and more frequently when infected focal termites were visibly ill and moribund (i.e. viable blastospores at 15 h). All focal termites were alive at the time of cannibalism (beginning < 24 h after injection), tallying with the survival of injected individual termites when kept in isolation, where blastospore-induced death began between 24 and 48 h post injection (Supplementary Fig. S5). Control focal termites were left unharmed.

In survival analyses, significant differences were detected between viable blastospore and Ringer-injected control treatments (2 h, Blastospores+ vs Ringer *z* = 3.312 *P* = 0.0028; 8 h, Blastospores+ vs Ringer *z* = 3.226 *P* = 0.0038; 12 h, Blastospores + vs Ringer *z* = 2.808 *P* = 0.0150; 15 h, Blastospores+ vs Ringer *z*= 4.822 *P* < 0.0001). Cannibalism was observed in all dead-blastospore treatments and was significantly higher than Ringer-injected controls for all incubation time points (2 h, Blastospores− vs Ringer *z* = 3.114 *P* = 0.0055; 8 h, Blastospores− vs Ringer *z* = 2.397 *P* = 0.0497; 12 h, Blastospores− vs Ringer *z* = 2.946 *P* = 0.0097; 15 h, Blastospores− vs Ringer *z* = 2.684 *P* = 0.0218) (Fig. 3B, and Supplementary Table S2). Except for 1 individual in the 15 h/Ringer treatment, cannibalism was not observed for any Ringer-injected focal termites (Fig. 3A, and Supplementary Table S2).

In comparisons of viable and dead blastospore-injected treatments, the probability of remaining unharmed differed significantly only at the 15 h incubation time point (15 h, Blastospores+ vs Blastospores− *z* = 4.054 *P* < 0.0002). At this incubation time point, focal termites injected with viable blastospores were quickly dismembered, whereas dead blastospore-injected individuals were cannibalized more slowly. Even so, these individuals were completely cannibalized by 24 h after introduction (Fig. 3B). Complete elimination of focal individuals quantified at 24 h after introduction into experimental colonies was significantly higher for both viable and dead blastospore treatments compared to controls: (time in hours after injection) (26 h, Blastospores− vs Ringer *P* = 0.0032, Blastospores+ vs Ringer *P* = 0.0018; 32 h, Blastospores− vs Ringer *P* = 0.0044, Blastospores+ vs Ringer *P* = 0.0023; 36 h, Blastospores− vs Ringer *P* = 0.0018, Blastospores+ vs Ringer *P* = 0.0012; 39 h, Blastospores− vs Ringer *P* = 0.0012, Blastospores+ vs Ringer *P* = 0.0012). Close to 100% of all focal individuals were eliminated in both viable and dead blastospore treatments. This is contrasted with the survival of the majority of termites following dead blastospore injection when kept individually (Supplementary Fig. S5). The percentage of eliminated focal individuals quantified at 24 h did not significantly differ between viable and dead blastospore-injected individuals (26 h, Blastospores+ vs Blastospores− *P* = 0.5150; 32 h, Blastospores+ vs Blastospores− *P* = 0.2244; 36 h, Blastospores+ vs Blastospores− *P* = 1; 39 h, Blastospores+ vs Blastospores− *P* = 1) (Fig. 3B).

In the blastospore filtrate experiment, significantly increased cannibalism was observed in both the dead blastospore and filtrate-injected treatments compared to the control (Blastospores− vs Ringer z = 3.587 *P* = 0.0008; Filtrate vs Ringer z = 3.339 *P* = 0.0021) (Supplementary Table S4 and Supplementary Fig. S10). Termites challenged with dead blastospores were generally cannibalized at a higher rate, although the amount of cannibalism and overall survival probability was not significantly different between dead blastospore and filtrate injection (Supplementary Table S4).

### Moribund termites display altered cuticular hydrocarbon profiles

**Figure 4:**
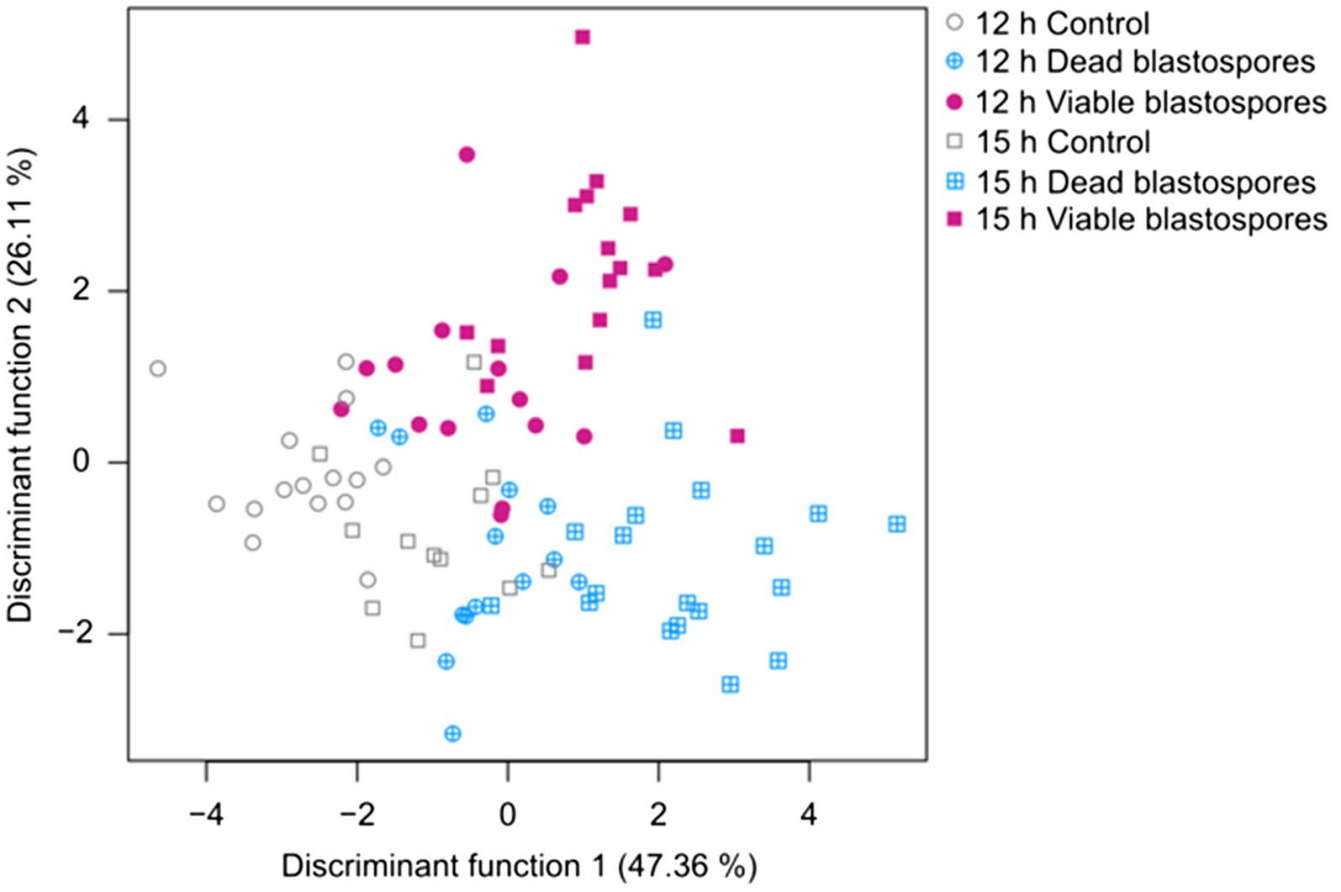
Linear discriminant analysis (LDA) based on 90 cuticular hydrocarbon (CHC) extracts from worker pools (3 individuals each) taken from 3 different *R. flavipes* colonies. The workers were treated with viable and dead blastospores, and Ringer solution as a control, and they were freeze-killed 12 or 15 h after injection. Overall differentiation was significant according to the different treatments (Wilk’s λ < 0.05, p < 0.05), the respective contributions of the two discriminant functions to the total variation are indicated in percentages. Different colors and symbols indicate different treatments and time points, respectively. Different colony affiliations are not indicated due to them not resulting in consistently discernable differentiation.

**Figure 5:**
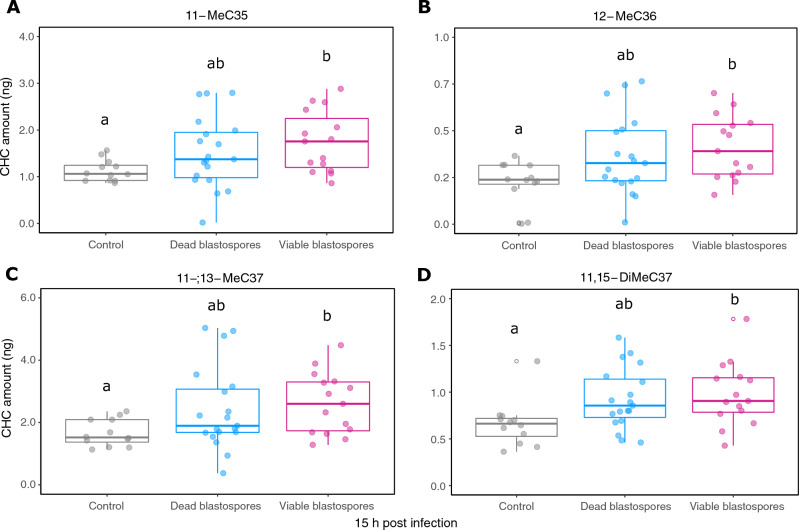
Quantitative comparison of a subset of CHC compounds (three mono- and one di-methyl-branched alkane) 15 h post injection of viable and dead blastospores, or Ringer solution as a control. CHC amounts were measured in ng as individual equivalents from extracts of worker pools (3 individuals each) taken from 3 different *R. flavipes* colonies. The absolute quantities of each of these compounds were significantly increased 15 h after injection of active blastospores as compared to the Ringer control (Benjamini–Hochberg corrected Mann–Whitney U tests). Although injection with inactive blastospores did not lead to significant increases after 15 h, an upward trend is observable.

A total of 90 individual equivalents of CHC extracts from worker pools (3 individuals each) taken from 3 different *R. flavipes* colonies were pre-defined into six groups for a discriminant analysis (DA) according to the different treatments: 12 and 15 h after injection of viable and dead blastospores and Ringer’s solution as a control, respectively (see above). Overall differentiation of the CHC profiles was significant according to the different treatments (Wilk’s λ < 0.05, *P* < 0.05). Discriminant function 1 accounted for 47.36% and discriminant function 2 for 26.11% of the total variation, amounting to 73.47% of total variance explained by the first two functions (Fig. 4). Concerning the variations in specific CHC compounds according to treatments, 15 h after injection, four methyl-branched alkanes consistently displayed significantly higher quantities in the viable blastospore treatment versus the control (*P* < 0.05, Benjamini–Hochberg corrected Mann–Whitney U tests, Fig. 5). These compounds were 11-MeC35, 12-MeC36, 11-; 13-MeC37, and 11,15-DiMeC37. Injections with dead blastospores also led to a visible, albeit non-significant increase in overall quantities of these particular compounds compared to the controls. An overview of all identified CHCs including their respective absolute quantities per treatment is given in Table 1.

**Table 1:** Retention indices (RI), compound identifications and the respective absolute quantities as well as standard deviations (in ng) for all CHCs compared between treatments of *R. flavipes* workers: 12 and 15 h post injection of viable and dead blastospores and Ringer solution as a control, respectively.

RI	Compound ID	12 h dead blastospores	12 h viable blastospores	12 h control	15 h dead blastospores	15 h viable blastospores	15 h control
2207	*n*-C22	0.12 ± 0.07	0.11 ± 0.08	0.09 ± 0.08	0.12 ± 0.06	0.15 ± 0.1	0.08 ± 0.05
2270	3-MeC22	0.09 ± 0.26	0.04 ± 0.05	0.02 ± 0.02	0.03 ± 0.02	0.04 ± 0.04	0.1 ± 0.27
2280	X-C23:1	1.15 ± 0.69	0.96 ± 0.59	0.84 ± 0.69	1.4 ± 0.71	1.64 ± 1.35	0.86 ± 0.66
2287	X'-C23:1	0.07 ± 0.03	0.07 ± 0.04	0.05 ± 0.03	0.09 ± 0.03	0.11 ± 0.06	0.05 ± 0.02
2310	*n*-C23	9.2 ± 5.76	8.28 ± 5.93	8.04 ± 6.3	9.77 ± 4.21	11.69 ± 7.68	7.34 ± 4.79
2343	11-; 13-MeC23	0.77 ± 0.47	0.66 ± 0.41	0.59 ± 0.44	0.92 ± 0.42	1.04 ± 0.72	0.63 ± 0.43
2371	4-MeC23	1.61 ± 1.08	1.54 ± 1.09	1.27 ± 0.87	1.87 ± 0.84	2.21 ± 1.46	1.33 ± 0.84
2380	C24:1	0.57 ± 0.33	0.53 ± 0.37	0.43 ± 0.3	0.67 ± 0.23	0.82 ± 0.53	0.45 ± 0.29
2409	*n*-C24	5.66 ± 2.97	5.23 ± 3.11	5.03 ± 2.89	5.93 ± 1.61	6.89 ± 3.6	4.85 ± 2.28
2442	12-; 14-MeC24	1.45 ± 0.77	1.36 ± 0.79	1.12 ± 0.68	1.82 ± 0.65	1.96 ± 1	1.27 ± 0.73
2463	X-C25:1	0.1 ± 0.04	0.12 ± 0.04	0.1 ± 0.05	0.12 ± 0.04	0.16 ± 0.08	0.1 ± 0.03
2473	4-MeC24	16.46 ± 8.66	16.46 ± 9.71	13.92 ± 7.83	18.29 ± 5.39	22.33 ± 11.84	13.99 ± 6.49
2483	X'-C25:1	9.58 ± 5.23	9.19 ± 6.17	7.3 ± 4.86	11.7 ± 4.15	14.79 ± 8.41	7.7 ± 4.07
2483	X-C25:2	6.02 ± 3.87	5.97 ± 4.53	4.49 ± 2.77	6.97 ± 3.69	8.9 ± 6.29	4.64 ± 3.12
2509	*n*-C25	25.88 ± 12.72	24.05 ± 13.28	23.74 ± 12.34	27 ± 6.45	31.66 ± 15.7	22.96 ± 9.55
2542	9-; 11-; 13-; 15-MeC25	21.06 ± 11.28	19.47 ± 11.1	16.07 ± 9.25	24.94 ± 7.95	27.99 ± 14.37	18.62 ± 10.35
2555	X'-C25:2	3.29 ± 1.75	3.12 ± 1.81	2.72 ± 2.17	3.59 ± 1.61	4.74 ± 3.51	2.48 ± 1.39
2557	5-MeC25	2.58 ± 1.87	2.14 ± 1.68	1.68 ± 1.16	2.96 ± 1.99	4.09 ± 3.49	2.11 ± 1.52
2575	4-MeC25	3.37 ± 1.59	3.22 ± 1.59	2.7 ± 1.48	3.89 ± 1.05	4.67 ± 2.17	2.78 ± 1.19
2577	X''-C25:2	3.19 ± 1.95	3.15 ± 2.31	2.7 ± 1.5	3.66 ± 1.53	4.37 ± 2.72	2.86 ± 1.63
2581	3-MeC25	6.34 ± 3.15	6.24 ± 3.48	5.41 ± 3.11	7.06 ± 2.21	8.7 ± 4.72	5.59 ± 2.89
2641	12-; 14-; 16-MeC26	0.69 ± 0.45	0.63 ± 0.44	0.49 ± 0.31	0.73 ± 0.36	0.9 ± 0.51	0.58 ± 0.33
2651	6-MeC26	0.21 ± 0.09	0.2 ± 0.1	0.18 ± 0.12	0.25 ± 0.11	0.34 ± 0.28	0.2 ± 0.12
2669	4-MeC26	0.78 ± 0.42	0.71 ± 0.51	0.65 ± 0.39	0.89 ± 0.34	1.05 ± 0.56	0.69 ± 0.36
2681	3-MeC26 + X-C27:1	0.15 ± 0.1	0.14 ± 0.08	0.23 ± 0.2	0.18 ± 0.11	0.27 ± 0.17	0.14 ± 0.09
2711	*n*-C27	0.22 ± 0.11	0.18 ± 0.12	0.47 ± 0.36	0.28 ± 0.37	0.76 ± 2.11	0.28 ± 0.24
2742	11-; 13-; 15-MeC27	0.11 ± 0.05	0.08 ± 0.05	0.07 ± 0.04	0.12 ± 0.05	0.14 ± 0.07	0.1 ± 0.06
2744	X'-C27:1	0.29 ± 0.17	0.25 ± 0.17	0.21 ± 0.2	0.29 ± 0.18	0.45 ± 0.4	0.21 ± 0.15
2756	X'-C27:2	0.11 ± 0.05	0.09 ± 0.07	0.09 ± 0.05	0.13 ± 0.07	0.14 ± 0.06	0.11 ± 0.04
2792	5,17-diMeC27	0.06 ± 0.03	0.05 ± 0.03	0.05 ± 0.04	0.07 ± 0.04	0.09 ± 0.11	0.05 ± 0.04
3531	**11-MeC35**	1.33 ± 0.62	1.44 ± 0.8	1.07 ± 0.41	1.52 ± 0.76	**1.75 ± 0.65**	**1.13 ± 0.23**
3629	**12-MeC36**	0.32 ± 0.15	0.34 ± 0.21	0.24 ± 0.12	0.37 ± 0.21	**0.41 ± 0.17**	**0.22 ± 0.11**
3729	**11-; 13-MeC37**	2.12 ± 0.9	2.21 ± 1.16	1.7 ± 0.6	2.42 ± 1.33	**2.62 ± 0.98**	**1.66 ± 0.43**
3751	**11,15-diMeC37**	0.77 ± 0.32	0.79 ± 0.38	0.6 ± 0.18	0.89 ± 0.38	**0.96 ± 0.33**	**0.61 ± 0.25**
3774	5,15-; 5,17-diMeC37	0.91 ± 0.47	0.93 ± 0.53	0.7 ± 0.22	1.07 ± 0.56	1.11 ± 0.48	0.67 ± 0.24
3927	13-MeC39	0.81 ± 0.39	0.83 ± 0.51	0.62 ± 0.22	0.94 ± 0.62	0.98 ± 0.46	0.61 ± 0.25
3949	11,15-diMeC39	0.62 ± 0.31	0.63 ± 0.35	0.49 ± 0.12	0.73 ± 0.39	0.73 ± 0.3	0.52 ± 0.1
3973	5,15-; 5,17-diMeC39	0.54 ± 0.28	0.56 ± 0.34	0.4 ± 0.13	0.65 ± 0.39	0.63 ± 0.31	0.43 ± 0.1

Retention indices were calculated according to the position of detected *n*-alkanes in our samples and, where not available, with a C21-40* n*-alkane standard run under the same conditions. Significantly different compound quantities are indicated in bold, accessed through Benjamini–Hochberg corrected Mann–Whitney U tests.

## Discussion

Our study shows that the collective response towards pathogen-exposed termites varies according to the severity and timing of internal challenge, and furthermore, that these are associated with alterations to the surface chemistry of infected individuals. Notably, we demonstrate that the decision to eliminate an individual via cannibalism is triggered at an early stage after pathogen exposure, independent of pathogen viability or even terminal disease status.

Individuals that were moribund at 15 h following viable blastospore injection were cannibalized at a higher rate than individuals injected with an equivalent dose of dead blastospores or a control solution. These observations complement previous findings from fungus-exposed individuals that were cannibalized when *R. flavipes* termites were close to death^17^. Surprisingly, however, we observed increased cannibalism in response to both viable and dead blastospore challenge as early as 2 h post injection (p.i.), when termites did not yet display overt signs of illness. This means that cannibalism is triggered in response to individuals that are not necessarily terminally ill. This can be seen in focal termites injected with dead blastospores, which induced 40% mortality at the individual level yet almost all were eventually cannibalized by 24 h p.i. when placed inside experimental colonies. Dead blastospore-injected termites did not display severe signs of sickness even at later experimental time points, and generally died much later than viable blastospore-injected individuals when kept in isolation. A similar pattern was observed when termites were injected with a dead blastospore solution lacking both fungal cells and intact destruxins, showing that even subcellular fungal components lacking active toxins are sufficient to elicit a strong cannibalism response. The cause of dead blastospore induced death remains unclear but represents a worthy topic for future investigation.

These results reveal that cannibalism is triggered before the onset of moribundity. This could reflect the need to react rapidly to the natural course of disease progression caused by entomopathogenic fungi such as *Metarhizium*, indicating that *R. flavipes* might employ a conservative strategy towards individuals displaying signs of internal fungal infection. *Metarhizium* deploys a multi-component infection strategy to kill the host and complete the infection cycle, including toxin release after the fungus breaches the cuticular barrier^49^, so once this stage has been reached, host death is likely unavoidable under natural circumstances. There are several points during internal infection that could activate endogenous signalling pathways, which in turn may communicate a change in disease status to interacting nestmates. The first may be as soon as when blastospores have gained access to the hemocoel, as this could feasibly be linked to the rapid activation of innate immune signalling pathways. The second stage could be associated with fungal acquisition of nutrients for growth and reproduction, during which time termite hosts gradually begin to show visible signs of sickness (from 12 h p.i.). Similar symptoms have been described from the higher termite *Nasutitermes exitiosus*^53^. Our results indicate that elimination cues are perceived by nestmates from an early stage following cuticular breach, but also that reinforcement cues may be generated that correlate with the progression and severity of infection. Correlated with the phase of fungal proliferation, is the ramping up of toxin biosynthesis^49,54,57,59,64^ which likely accelerates host death^46,53,57^. Blastospores might evade innate insect immune responses through reduction of the number of pathogen‐associated molecular patterns (PAMPs) by camouflaging or β-glucan modification^65,66^, but in general their interaction with the termite immune system is poorly understood, and represents an interesting target for future research.

With respect to inactivated blastospores, these harbour several components that could activate the immune system of the host, including denatured cellular contents and cell wall constituents. One factor to consider is that heat-inactivated blastospores did not contain intact destruxins. Such missing factors could represent an important form of danger signal, either via direct interaction with signalling receptors, or indirectly via their negative influence on host cells, which may then produce signals of damage^67^. It is possible that the faster cannibalism response to viable blastospore-injected termites is linked to the presence and active synthesis of fungal virulence factors such as destruxins, although this also requires further testing.

We hypothesized that activation of endogenous host pathways would result in external changes that may be perceptible by interacting nestmates. We examined cuticular hydrocarbon (CHC) profiles of internal pathogen-challenged individuals to understand whether they could serve as chemical cues indicating infection. Injections with both viable and inactivated blastospores shifted overall CHC profiles, with a trend towards more clear differentiation after a longer incubation time. We detected significant increases in four methyl-branched CHCs 15 h after injection with viable blastospores compared to control-injected individuals. Interestingly, these four compounds are structurally related, long-chained mono- and di-methyl-branched alkanes with a similar chain length range (C35-C37) and internal methyl-branches at positions 11, 12, 13 and 15. A trend towards an increase in these compounds was also clearly discernible albeit non-significant in individuals injected with dead blastospores. These results indicate a common pattern for encoding and conveying a chemical cue associated with infection status, which would be in line with the high potential of methyl-branched alkanes to encode chemical information^68–70^. However, whether these compounds are directly responsible for eliciting the observed cannibalism response requires further experimental validation. Similar findings have been reported in challenged *Lasius* ants where infection has been linked with changes in CHCs of a similar chain length range (C33-35), albeit of non-methyl branched compounds^19^ whereas in *Myrmica* ants, the relative proportion of straight-chain alkanes increased upon infection while methyl-branched alkanes decreased^34^. Studies reporting cuticular profile changes associated with disease have also been carried out in honeybees^29,32,35^. In line with these findings, our study points to host condition-dependent cues as playing an important role, with elimination behaviors potentially being activated when changes in CHC compounds exceed a given quantitative threshold^71^. Our observations of increased amounts of long-chained mono- and di-methyl-branched alkanes over time following viable blastospore injection, as well as a trend towards higher quantities of these compounds in dead blastospore injected termites, may be consistent with a threshold hypothesis.

With respect to other behaviors, in comparison to previous work employing conidia on the termite cuticle^17,18,23,40,42,57,72,73^, our observed rates of allogrooming are lower across comparable time points, suggesting that direct contact with external pathogens are more important in generating an allogrooming response. The benefits of allogrooming for enhancing group survival in the presence of external pathogens are well documented^14,40–42^, which makes intuitive sense as allogrooming can remove potentially infectious microbes on the surface before they breach the cuticle. The general absence of burial in our experiments supports the idea that termites preferentially eliminate harmful individuals through cannibalism^47,74^, although burial may be a more important defensive mechanism for older or decomposing cadavers^74–76^.

Overall, our results show that termites are capable of readily detecting and eliminating individuals displaying signs of internal fungal challenge. Our findings also indicate that elimination response behaviors are triggered before individuals enter a terminal disease state, and furthermore, that individuals that could theoretically recover will nonetheless be cannibalized by their nestmates. These findings may reflect the need for a vigilant social immune system that has evolved to recognize early signs of a typically highly virulent fungal infection. In terms of the care-kill paradigm, internal challenge with fungal blastospores ultimately appears to be associated with a kill response. Trade-offs in immune system vigilance have been explored in the context of beneficial versus pathogenic microbiota^77^ and similar trade-offs may also operate with regards to social immunity. Here, the level of social immune vigilance might be determined by pathogen hazard on the one hand, and cost of social immunity (damage to the colony due to elimination of constituent members as well as risk to the survival of intervening nestmates) on the other. In comparisons across species, factors such as colony size and maturity, as well as individual age could play a role in shaping this trade-off. As in individual immunity, a positive correlation between pathogen hazard and social immunity vigilance may be expected. It is perhaps unsurprising that *R. flavipes* does not differentiate between viable and inactivated blastospores or associated moribundity effects arising from an internal fungal challenge, because such a situation would not arise under natural conditions. Although we have not experimentally verified the precise cuticular chemical cues or underlying mechanisms used to trigger elimination, we speculate that they may involve crosstalk between immune and other physiological processes such as metabolism and the regulation of biosynthetic pathways involved in cuticular chemistry variation. Expansion of chemical analyses to other volatile compound classes such as alcohols (e.g. Butanol, 2,3-Butanediol) and ketones (e.g. 2-Pentanone) released by entomopathogenic fungi^78,79^ may also be warranted. Another interesting question to consider is whether modification of the cuticular profile in pathogen-challenged individuals is sufficient to induce antifungal immune responses in interacting nestmates^19,29,32,35,80^. Other targets for future work could include functional experiments to test which specific compounds, chemical combinations, or general alterations in overall CHC abundance are sufficient to trigger changes in social immunity.

## Methods

### Termites

Six *R. flavipes* colonies (1-6) were used in these experiments. Additional information regarding host organisms is given in the supplementary material.

### Entomopathogenic fungus

We used the semelparous fungal entomopathogen *Metarhizium robertsii* (DSM 1490), previously classified as *Metarhizium anisopliae* by the German Collection of Microorganisms and Cell Cultures GmbH (DSMZ) (https://www.dsmz.de/collection/catalogue/details/culture/DSM-1490). *M. anisopliae* and *M. robertsii* are closely-related obligate-killing entomopathogens of *R. flavipes*, and are detected in soils in close proximity to colonies^81^. Details on fungal conidia germination and harvesting methods are given in the supplementary material.

To prepare cultured blastospores, we added 1 mL of a 1 × 10^8^ conidia/mL suspension to 100 mL of two different liquid media in 300 mL Erlenmeyer flasks: (i) 40 g/L yeast extract, 80 g/L glucose and 0.1% Tween 80; (ii) 40 g/L yeast extract, 40 g/L glucose, 30 g/L corn steep liquor and 0.1% Tween 80^82^. Erlenmeyer flasks were incubated in a shaking incubator for 3 days (exponential phase) at 25 °C and 290 rpm. Blastospores were then harvested by combining the two cultured media and filtering them through two layers of a sterile miracloth (Merck KGaA, Darmstadt, Germany) to remove the mycelia. The filtrate was centrifuged for 5 min at 2000*g* at 4 °C and the pellet containing the blastospores was washed and resuspended three times in Ringer’s ¼ solution. As with conidia, blastospore concentrations were determined in a Thoma counting chamber and adjusted to 5 × 10^8^ blastospores/mL. Experimental injections consisted of two pathogen and one control treatment. The two pathogen treatments were: viable challenge, using viable blastospores (5 × 10^8^ blastospores/mL viable blastospores; 96% germination rate at 10 h) or inactivated challenge, using dead blastospores (5 × 10^8^ heat-inactivated blastospores/mL; 0% germination). The control treatment consisted of sterile Ringer’s ¼ solution only. To prepare the dead blastospore treatment, an aliquot of the viable blastospore suspension was autoclaved. Germination was assessed on two PDA plates streaked with 50 μL of the autoclaved blastospore solution, followed by incubation in the dark at 25 °C for 24 h. No fungal growth was observed in the dead blastospore treatment. Blastospores were considered to have germinated when an elongating germ tube of any size was visible at 200–400× magnification^83^.

### Preparation of experimental colonies

Experimental *R. flavipes* colonies were set up inside Petri dishes as described elsewhere^17^ and as summarized in the supplementary material.

### Injection of focal termites

We marked focal termites with Nile blue, a fat-soluble stain that has been used in several studies to mark termites and has not been reported to affect termite behavior^17,84^. It dyes the termites blue, making them easily distinguishable from their otherwise colorless nestmates. Nile blue dyeing was carried out following a rapid method for marking termites as described previously^17,85^. Each focal termite was anaesthetized with CO_2_, then injected with 41.4 nL of either viable blastospores, dead blastospores, Ringer’s solution or fungal filtrate directly into the hemocoel using a Nanoject II (Drummond Scientific Company, USA). The needle was inserted into the side of the thorax. With this method, the first stages of the *Metarhizium* infection process, that is, adhesion, germination of fungal conidia and cuticle penetration^86,87^ were bypassed. As described above, this allowed us to test the effect of indirect cues triggered by internal pathogen challenge. As expected from previous studies^49,55,83^, germination and killing rates are indeed faster when using blastospores as compared to conidia (Supplementary Figs. S1, S11)^17^. By carrying out further experiments with dead blastospore and fungal filtrate treatments, we were able to test whether active infection and moribundity were necessary to induce cannibalism.

Injected focal termites were kept individually in small Petri dishes (35 Ø mm), each containing a Pall cellulose pad moistened with 1 mL of distilled water and then incubated for either 2, 8, 12 or 15 h at 27 °C and 70% humidity post injection (p.i.) before use in behavioral observation experiments. Representative images and videos of focal termite and nestmate interactions inside experimental colonies were captured with a smartphone (Samsung galaxy S7) and adjusted for brightness and size (Adobe Photoshop, Adobe Systems, San Jose, California) to highlight different social immune behaviors (Supplementary Videos). Pathogen dose and incubation times were determined based on blastospore germination and initial survival assays of termites injected with different concentrations of *M. robertsii* blastospores (Supplementary Figs. S1, S11). The effect of the selected dose of viable and dead blastospores on termite survival when kept individually was examined by injecting 180 *R. flavipes* workers from three different colonies, each assigned to one of the following treatments: (i) 60 individuals: Viable blastospores (injected 5 × 10^8^ blastospores/mL); (ii) 60 individuals: Dead blastospores (injected 5 × 10^8^ heat-killed blastospores/mL); (iii) 60 individuals: control (injected Ringer’s solution). Survival of each individual was recorded every 12 h for a total of 240 h (Supplementary Fig. S5).

### Experimental design

Termites injected with the selected dose of viable blastospores were moribund by 16 h p.i., with death occurring between 22 and 26 h (Supplementary Figs. S5, S11). Knowledge of the germination and infection timeline in *R. flavipes* (Supplementary Figs. S1, S2 and Table S5) was used to select four incubation time points for behavioral observations: (i) 2 h: blastospores have gained access to the hemocoel but have not yet germinated; (ii) 8 h: blastospores have germinated but the host appears healthy; (iii) 12 h: the host shows first signs of sickness, as indicated by slower movements; and (iv) 15 h: the host is moribund (limited to no mobility). For individuals injected with dead blastospores, symptoms do not appear until 22 h p.i., and only among individuals that subsequently died (Supplementary Table S5).

#### Blastospore experiment

Focal termites were injected with either a suspension containing 5 × 10^8^ viable blastospores per mL, a suspension containing 5 × 10^8^ dead blastospores per mL, or Ringer’s solution as a control. Following experimental challenge with *M. robertsii* blastospores, focal termites were kept individually for the pre-assigned incubation times (2, 8, 12 and 15 h) prior to introduction into experimental colonies. For each of the four incubation time points, we conducted 24 replicates from 5 independent colonies for each treatment. Colonies 5 and 6 contributed 3 plate replicates per incubation time point and treatment, while colonies 1, 3 and 4 each contributed 6. Data for colonies 3, 4, 5 and 6 were collected over 2 experimental repeats (with colony 6 replacing 5 in the second repeat) while data for colony 1 were collected from a third experimental repeat. Plates from each incubation time were randomly assigned to one of the three treatment groups after the 15-day colony establishment period had elapsed (Total N = 288 plates). Randomisation was achieved by using a random number generator to produce double-digit number combinations, where the first digit corresponded to a colony and the second to one of the treatments. Challenged and control termites were introduced individually into the experimental colonies, which were then resealed with parafilm. The observation period began immediately after the last experimental colony was sealed.

#### Blastospore filtrate experiment

To further narrow down the pathogen components that may be responsible for inducing termite social immunity, we tested the effect of heat-inactivated fungal filtrate (lacking blastospores) on nestmate behavior. Filtrates consisted of the final Ringer’s ¼ solution used to wash dead blastospores, and therefore contain subcellular components and metabolites but not larger blastospore-sized structural components, which were removed by filtration. Fifty-four experimental plates of *R. flavipes* from colonies 2, 3, and 5 (6 plates per colony) and the respective focal termites were randomly assigned to one of the three treatment groups (Ringer, dead blastospores, or fungal filtrate) after the 15-day colony establishment period had elapsed (N = 18 per treatment). Focal termites were injected either with a suspension containing dead blastospores, fungal filtrate or Ringer ‘s solution, which served as a control. In contrast to the blastospore experiment, challenged termites were all isolated for 12 h prior to introduction into the experimental colonies and behaviors were recorded at different time points, as described below.

#### Behavioral recording

In the blastospore experiment, after 2, 8, 12, or 15 h of incubation, injected focal termites were added individually to the Petri dish colonies. To minimize vibrational stimuli that *R. flavipes* are known to be sensitive to^88^, Petri dishes were not opened or moved after sealing. This process took approximately 10 min, and the observation period began immediately after the last dish was sealed. For observations, we adopted a scan sampling method used previously^17^. In the blastospore experiment, we performed scan sampling of each Petri dish nest every 5 min for a total of 3 h^89^. For the blastospore filtrate experiment, behavioral responses were recorded later, due to the delayed onset of cannibalism in the dead blastospore treatment. Here scan sampling was carried out for 5 min every 2 h at 12, 15, 18 and 21 h post-injection. Scanning typically took around 1 min. When necessary, a magnifying glass was used (up to 3× magnification) to better distinguish between observed behaviors and a Samsung S7 smartphone was employed as a digital voice recorder. Observations were performed under constant overhead light at 27 °C and 70% humidity. To minimize observer bias, observations were carried out blind by randomly assigning a treatment to each Petri dish nest prior to the observation period as mentioned in the above section. In both experiments, focal termites were counted 24 h after introduction into the groups to quantify survival. Behaviors of interest were defined a priori and classified into visually distinguishable and non-overlapping categories relevant to social immunity. We focused on five different behavioral categories:

- *Groomed by n (gb):* Focal termite is being groomed by N nestmates with no evidence of biting.
- *Cannibalism (c):* Focal termite is being bitten by one or more nestmates and/or the focal termite body is no longer intact.
- *Buried (b):* Focal termite has had pieces of paper or faeces placed on it. The termite may still be alive. Note that this behavior was not observed and was therefore excluded from the analysis.
- *Not visible (nv):* Focal termite is in a part of the nest where it cannot be observed.
- *Other (o):* Focal termite is alive, intact, and unburied, but nestmates are not interacting with it. This reflects behavioral states unrelated to social immunity.

### Destruxin analysis

Blastospore cultures were prepared as described above, except that Czapek Dox liquid medium was used instead (modified, Oxoid, England)^90^. Viable blastospore cultures were subjected to vacuum filtration (Thermo Scientific™ Nalgene™ Rapid-Flow™, USA) to remove all fungal cells. Dead blastospores were generated as described above. Autoclaved blastospores in Ringer’s solution (as per the dead blastospore treatment) were then subjected to vacuum filtration to remove remaining fungal cells and used in the subsequent ultra-high pressure liquid chromatography coupled with tandem mass spectrometry (UHPLC-MS/MS) analysis for confirmation of the presence of fungal destruxins. For this purpose, a Synapt G2-S HDMS, equipped with an Acquity UPLC system (Waters Co., Milford, MA, USA) was used. The conditions of the mass spectrometer were optimized for small peptides, using the following settings: (+)-ESI in sensitivity mode; capillary voltage: 3.3 kV, sample cone voltage: 40; source offset: 60; source temperature: 90 °C; desolvation temperature: 250 °C; cone gas flow: 1 L/h; desolvation gas flow: 500 L/h; nebulizer gas flow: 6 bar. UPLC grade solvents used throughout the analysis were provided by Biosolve BV, Valkenswaard, Netherlands. The UHPLC conditions were optimized to an injection volume of 7 µL; flow rate: 0.25 mL/min; eluents: A: water + 0.1% formic acid; B: acetonitrile + 0.1% formic acid; column thermostat: 40 °C; gradient: 0 min: 25% B, 10 min: 40% B, 13 min: 40% B, 15 min: 95% B, 20 min: 95% B, 21 min: 25% B, 25 min: 25% B. This method was adopted following Taibon et al.^63^. Comparison with literature (retention times and fragmentation pattern) confirmed the identities of the destruxins as observed from the chromatograms^62,63^.

### Statistical analysis

Statistical analyses were carried out in R version 4.2.2 and RStudio version 2022.07.02.

#### Grooming

The amount of grooming (defined as the number of grooming states/total observed states) in each treatment was analysed by fitting a generalized linear mixed model (GLMM) to the data using the *glmer* function in the R package *lme4*^91^. A binomial error distribution was used to model proportion data^92^. For the blastospore experiment, models for each incubation time (2, 8, 12, 15 h) were composed of treatment and amount of cannibalism (defined as the number of cannibalism states/total observed states) as fixed effects. Petri dish nest ID and colony were used as nested random effects (Plate within colony). For the blastospore filtrate experiment, models were also constructed for each time point (12, 15, 18, 21 h) and contained treatment, amount of cannibalism and colony as fixed effects and Petri dish nest ID as a random effect. All models were compared using Akaike information criterion (*AIC*-function in R), which revealed the full model to be the best fit. Model assumptions were checked using the functions *simulateResiduals*, *testDispersion* and *testZeroInflation* contained within the *DHARMa* package^93^. We performed post hoc pairwise comparisons using the *glht* function from the *multcomp* package v1.4-10 with Tukey tests using a Bonferroni correction^94,95^.

#### Cannibalism

The *survfit*-function from the *survival* package^96^ was used to model the onset of cannibalistic behavior and the *ggsurvplot*-function from the *survminer* package^97^ was used to plot the data. Survival curves were compared using a mixed effects Cox model (*coxme function* from the *coxme* package^98^). For the blastospore experiment, models for each incubation time (2, 8, 12, 15 h) included treatment as a fixed effect, and Petri dish nest ID and colony as random effects. For the blastospore filtrate experiment, a single model included treatment and colony as fixed effects and Petri dish nest ID as a random effect. In the survival curve analyses, all control data was initially right-censored; in order to fit a mixed effects Cox model to the data, it was necessary to uncensor one arbitrarily-selected control replicate from each incubation time^17,24^. The *glht*-function was used to perform post-hoc pairwise comparisons using Tukey tests with Bonferroni corrections.

#### Mortality of focal termites at 24 h after introduction into experimental colonies

Following the main blastospore experiment, survival of focal termites was assessed 24 h after introduction into experimental colonies (corresponding to 26, 32, 36 and 39 h after injection for each incubation time point, respectively). Mortality rates were calculated for each treatment and time point. A Wilcoxon rank sum test was used to perform pairwise comparisons of mortality rate ranks between treatments, using a Bonferroni-correction to account for multiple comparisons.

### Scanning electron micrographs (SEMs) of blastospores

Representative SEMs of germinating blastospores on PDA media at different time points (0, 2, 4, 8, 12 and 15 h [Supplementary Fig. S2]) were captured to determine the in vitro germination rate. For SEM preparations, small and thin pieces of PDA with blastospores were mixed 1:1 with 2.5% glutaraldehyde in 0.1 M cacodylate buffer pH 7–7.2 for 60 min. After three 5–10 min washing steps with 0.1 M cacodylate buffer, the samples were fixed in 1% OsO_4_ in distilled water for 60 min and washed three times in distilled water for 5–10 min each. Then the blastospores were transferred into vessels covered with planktonic gauze (mesh-size 10 µm) and were dehydrated in ascending ethanol concentrations (30%, 50%, 70%, 90%, 2 × 100%, 2 × 100% absolutely water free) for 10–15 min each. Critical point drying with CO_2_ was performed with a Balzer CPD 030. The dried samples were transferred onto double-sided adhesive tape on specimen stubs before they were sputtered with gold in a Balzer SCD 040. Images were taken with a FEI Quanta 200 ESEM.

### Cuticular hydrocarbon analysis

Extractions of pools of three individual termites per treatment were performed in 2 mL glass vials (Agilent Technologies, Santa Clara, California, USA) on an orbital shaker (IKA KS 130 Basic, Staufen, Germany) for 10 min. Extracts were subsequently evaporated under a constant stream of gaseous carbon dioxide and then resuspended in 5 µL of a hexane solution containing 7.5 ng/μl dodecane (C12) as an internal standard. Following this, 3 μL of the resuspended extracts were injected in splitless mode with an automatic liquid sampler (ALS) (PAL RSI 120, CTC Analytics AG, Switzerland) into a gas-chromatograph (GC: 7890B) simultaneously coupled to a flame ionization detector (FID: G3440B) and a tandem mass spectrometer (MS/MS: 7010B, all provided by Agilent Technologies, Waldbronn, Germany). The system was equipped with a fused silica column (DB-5MS ultra inert; 30 m × 250 μm × 0.25 μm; Agilent J&W GC columns, Santa Clara, CA, USA) at a temperature of 300 °C with helium used as a carrier gas under a constant flow of 1.8 mL/min. The FID had a temperature of 300 °C and used nitrogen with a 20 mL/min flow rate as make-up gas and hydrogen with a 30 mL/min flow rate as fuel gas. The column was split at an auxiliary electronic pressure control (Aux EPC) module into an additional deactivated fused silica column piece (0.9 m × 250 μm) with a flow rate of 0.8 mL/min leading into the FID detector, and another deactivated fused silica column piece (1.33 m × 250 μm) at a flow rate of 1.33 mL/min into the mass spectrometer. The column temperature program started at 60 °C and was held for 5 min, increasing 20 °C/min up to 200 °C and then increasing 3 °C/min to the final temperature of 325 °C, held for 5 min.

CHC peak detection, integration, quantification and identification were all carried out with Quantitative Analysis MassHunter Workstation Software (Version B.09.00/Build 9.0.647.0, Agilent Technologies, Santa Clara, California, USA). CHCs were identified according to their retention indices, diagnostic ions, and mass spectra as provided by the total ion count (TIC) chromatograms, whereas their quantifications were achieved by the simultaneously obtained FID chromatograms, allowing for the combination of the best-suited method for hydrocarbon quantification (Agilent Technologies, Waldbronn, Germany, pers. comm.) with reliable compound identifications. Absolute CHC quantities (in ng) were obtained by standardizing all peak areas of the identified CHC compounds according to the concentration of the internal C12 standard, and additionally divided by three to obtain CHC amount equivalents per single individual. A discriminant analysis (DA) was performed with the R package MASS to test whether CHC profiles statistically differ between the investigated treatment groups and to visualize the degree of separation between them. Wilk's *λ* was calculated to measure the quality of the DA. Furthermore, quantitative differences between the treatments for each individual CHC compound were accessed with Benjamini–Hochberg corrected Man-Whitney U tests, and visualized with boxplots where significant differences were detected.

### Supplementary Information

Supplementary Information 1.

Supplementary Information 2.

## Data Availability

All data generated or analyzed during this study are included in this published article and its supplementary information files.
