# Supplementary material for: Defensive behavior is linked to altered surface chemistry following infection in a termite society
Source: Sci Rep. 2023 Nov 23;13:20606. doi: 10.1038/s41598-023-42947-9 (PMC10667546; doi:10.1038/s41598-023-42947-9)
Supplement: Supplementary file 1 — Supplementary Information 1. [file 41598_2023_42947_MOESM1_ESM.docx]

Supplementary Material for:

Defensive behavior is linked to altered surface chemistry following infection in a termite society

M. Alejandra Esparza-Mora^1,2^, Tilottama Mazumdar^1,2^, Shixiong Jiang^1,2^, Renate Radek^1^, Julian Nico Thiem^2^, Linshan Feng^2^, Vesta Petrašiūnaitė^2^, Ronald Banasiak^2^, Marek Golian^3^, Melanie Gleske^3^, Christophe Lucas^4^, Andreas Springer^5^, Jan Buellesbach^3^, Dino P. McMahon^1,2^

^1^ Institute of Biology, Freie Universität Berlin, Königin-Luise-Straße. 1-3, 14195 Berlin, Germany

^2^ Department for Materials and Environment, BAM Federal Institute for Materials Research and Testing, Unter den Eichen 87, 12205 Berlin, Germany

^3^ Institute for Evolution and Biodiversity, University of Münster, Hüfferstraße. 1, 48149 Münster, Germany

^4^ Institut de Recherche sur la Biologie de l’Insecte (UMR7261), CNRS – University of Tours, Tours, France

^5^ Core Facility BioSupraMol, Department of Biology, Chemistry and Pharmacy, Freie Universität Berlin, Takustraße. 3, 14195 Berlin, Germany

**Supplementary Methods**

**Termites**

The subterranean termite *Reticulitermes flavipes* is characterized by cryptic nesting habits, forming complex colonies whose diffuse nests and multiple feeding sites are connected by underground tunnels^1,2^. Six *R. flavipes* colonies were used in these experiments: colonies 1-6 (internally labelled as I, II, 5, 11+13, X and E, respectively). Pieces of wood containing dense aggregations of termites belonging to these colonies were collected from the field. Colonies 5, 11+13 and I were collected in Île d’Oléron, France, in 1994, 1999 and 2018 respectively and maintained in a dark room at 26 °C, 84% humidity. Colony E, X and II were collected in Soulac-sur-Mer, France in 2015 (colonies E and X) and 2018 (colony II) maintained in a dark room at 28 °C, 83% humidity. All colonies were kept in separate sheet metal tanks^3^, located at the Federal Institute for Materials Research and Testing (BAM) in Berlin, Germany, and had access to wood ad libitum as well as sufficient damp soil to burrow. Cardboard baits were used to isolate termites from their parental colonies^4^. Upon isolation, termites from the same colony were transferred to plastic boxes containing cellulose pads (Pall Corporation, Port Washington, USA) that had been moistened with tap water. Collected termites were kept at the same temperature as the parent colony until staining or transfer to experimental Petri dish nests.

**Entomopathogenic fungus**

We used the semelparous fungal entomopathogen *Metarhizium robertsii* (DSM 1490), previously classified as *Metarhizium anisopliae* by the German Collection of Microorganisms and Cell Cultures GmbH (DSMZ) (https://www.dsmz.de/collection/catalogue/details/culture/DSM-1490). *M. anisopliae* and *M. robertsii* are closely-related obligate-killing entomopathogens of *R. flavipes*, and are detected in soils in close proximity to colonies^5^. Conidia of the entomopathogenic fungus were stored at -70 °C. Prior to each experiment, the conidia were grown on potato dextrose agar (PDA) at 25 °C in darkness until sporulation. After 15 days of incubation the conidia were gently scraped off the plate with a cotton swab moistened with sterile 0.05% Tween 80 and suspended in sterile 0.05% Tween 80. The resulting conidia suspension was vortexed for 30 seconds and then filtered through a piece of sterile miracloth (Merck KGaA, Darmstadt, Germany) to remove hyphae and large clumps of conidia. The filtered conidia suspension was centrifuged for 10 minutes at 5000 *g* at 4 °C and the pellet was resuspended and washed three times with sterile 0.05% Tween 80. A BLAUBRANDR Thoma counting chamber (depth 0.1 mm; BRAND, Wertheim, Germany) was used to estimate the concentration of the conidia suspension. The concentration was adjusted to 1 x 10^8^ conidia/mL with sterile 0.05% Tween 80, aliquoted for ease of use, and and stored at 4 °C until further use (within 24 hours). Prior to use, germination rates of conidia were assessed and were over 95% in all cases. To assess germination, we streaked two PDA plates with 50 µL of the 1 × 10^8^ conidia/mL suspension and incubated them in the dark at 25 °C. After 21 hours of incubation, a minimum of 200 conidia per plate were evaluated for germination at 200-400× magnification. Conidia were considered germinated when the elongating germ tube was longer than the maximum conidial diameter^6^.

To prepare cultured blastospores, we added 1 mL of 1 x 10^8^ conidia/mL suspension to 100 mL quantities of two different liquid media in 300 mL Erlenmeyer flasks: (i) 40g/L yeast extract, 80g/L glucose and 0.1% Tween 80; (ii) 40g/L yeast extract, 40g/L glucose, 30g/L corn steep liquor and 0.1% Tween 80^7^. Erlenmeyer flasks were incubated on a shaker (290 rpm) in an incubator for 3 days (exponential phase) at 25 °C. Blastospores were then harvested by combining the two cultured media and filtering them through two layers of a sterile miracloth (Merck KGaA, Darmstadt, Germany) to remove the mycelia. The filtrate was centrifuged for 5 min at 2000*g* at 4 °C and the pellet containing the blastospores was washed and resuspended three times in Ringer´s ¼ solution. Just as with conidia, blastospore concentrations were determined in a Thoma counting chamber and adjusted to 5 x 10^8^ blastospores/mL. Experimental blastospore injections consisted of two pathogen and one control treatment. The two pathogen treatments were: viable challenge, using live blastospores (5 x 10^8^ blastospores/mL viable blastospores; 96% germination rate at 10 hours) or inactivated challenge, using dead blastospores (5 x 10^8^ heat-inactivated blastospores/mL; 0% germination). The control treatment consisted of sterile Ringer´s ¼ solution only. To prepare the dead blastospore treatment, an aliquot of the viable blastospore suspension was autoclaved. Germination was assessed on two PDA plates streaked with 50 μL of the autoclaved blastospore solution, followed by incubation in the dark at 25 °C for 24 hours. As expected, no fungal growth was observed. Blastospores were considered to have germinated when an elongating germ tube of any size was visible at 200-400× magnification^6^.

**Preparation of experimental colonies**

Experimental *R. flavipes* colonies were set up inside Petri dishes as described elsewhere^8^. Briefly, Petri dishes (94 × 16 mm) were prepared with two cellulose pads (45.5 Ø mm diameter, 0.9 mm thick) (Pall) placed on top of two thin Whatman No. 5 filter paper discs (47 Ø mm diameter, 0.2 mm thick). A standard microscope glass slide (76 × 26 mm) was then placed on top of the filter papers. To establish experimental colonies, we introduced a total of 49 healthy termites (not including the focal individual): 46 medium-to-large workers (3-5 mm body length), 2 representatives of the reproductive caste and 1 soldier. Fifty termites are a functional minimum suggested for lab-based experiments ^ref 3^ and as described previously, are sufficient to ensure colony establishment, and observe a natural repertoire of termite behaviours^8^. The paper was moistened with 3.5 mL of tap water prior to the introduction of the termites. Experimental nests were sealed with parafilm to maintain a high level of humidity within petri dishes and left in a dark room at 27 °C and 70% humidity for 15 days to enable the termites to establish tunnels under the glass slide. To ensure the clearest possible view into the nest, a cotton swab was used to clean the glass slide of any debris 24 hours prior to the behavioural experiment. The Petri dishes were resealed immediately after cleaning was done.

**Germination experiment**

To evaluate germination in vitro, 5 PDA plates were streaked with the 5 x 10^8^ blastospores/mL *Metarhizium robertsii* 1490 suspension, sealed with parafilm, and incubated in the dark at 25 °C. At 0, 2, 4, 6, 10 and 12 hours after inoculation, at least 100 blastospores per plate were evaluated for germination at 200-400x magnification using a phase contrast microscope. After 12 h, dense hyphal growth made it impossible to calculate the germination rate. Means and 95% confidence intervals were calculated and plotted using R version 4.2.2 and RStudio version 2022.07.02.


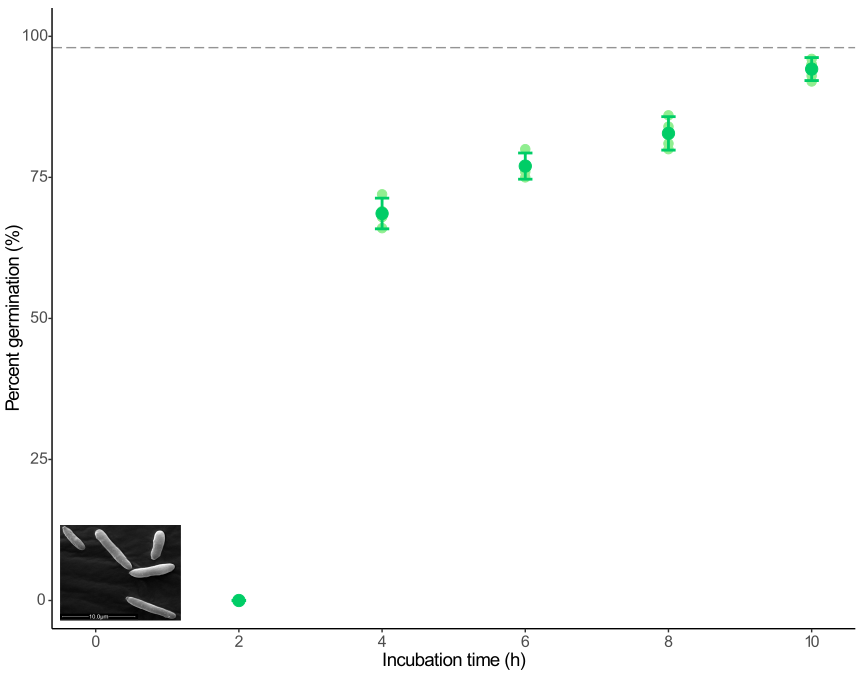


**Supplementary Figure S1**. Germination of blastospores on PDA plates. Larger dots represent means, smaller dots are individual data points, and bars show 95% confidence intervals.


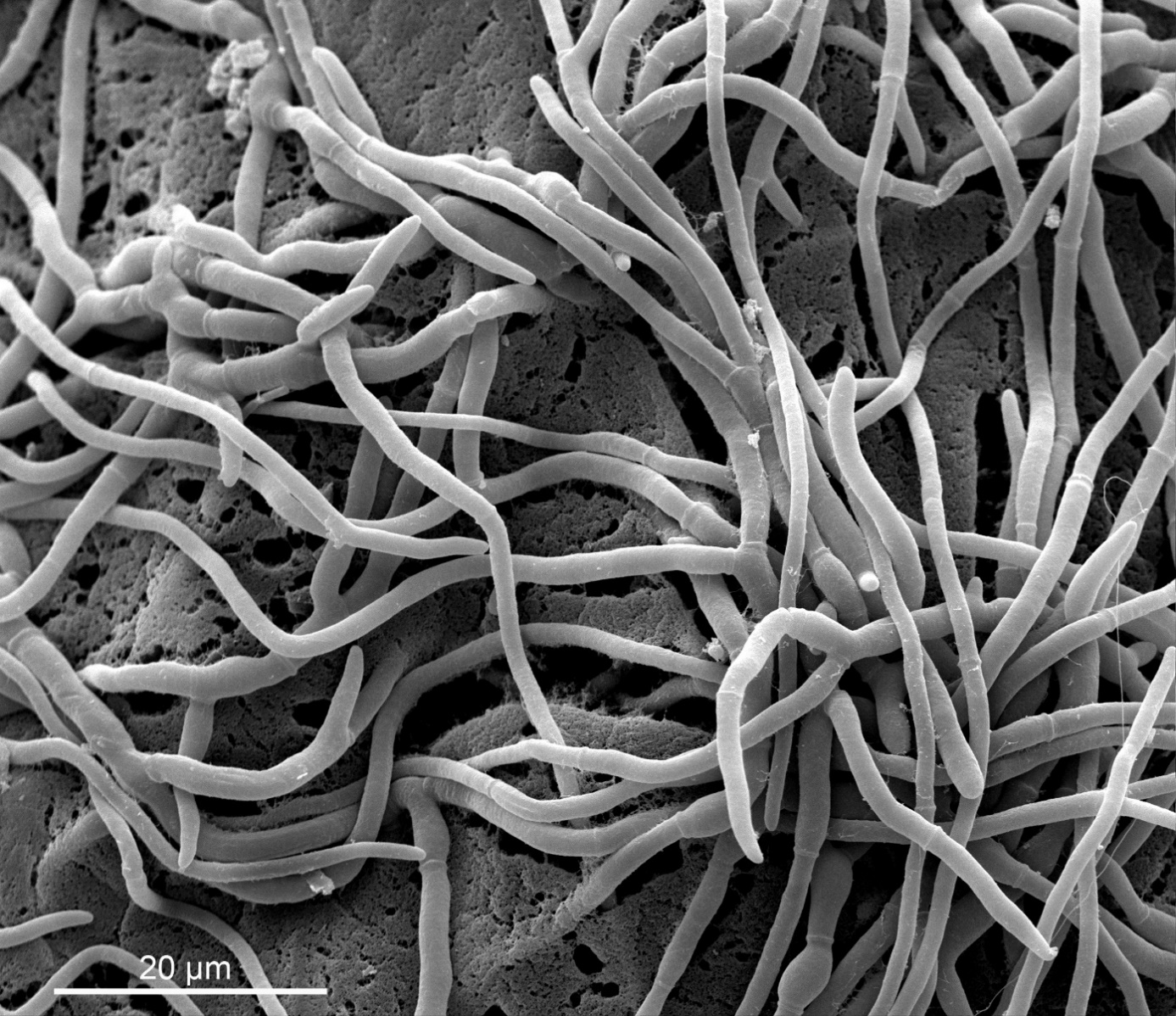


**Supplementary Figure S2.** SEM image of blastospore germination at 15 hours post-inoculation.

**
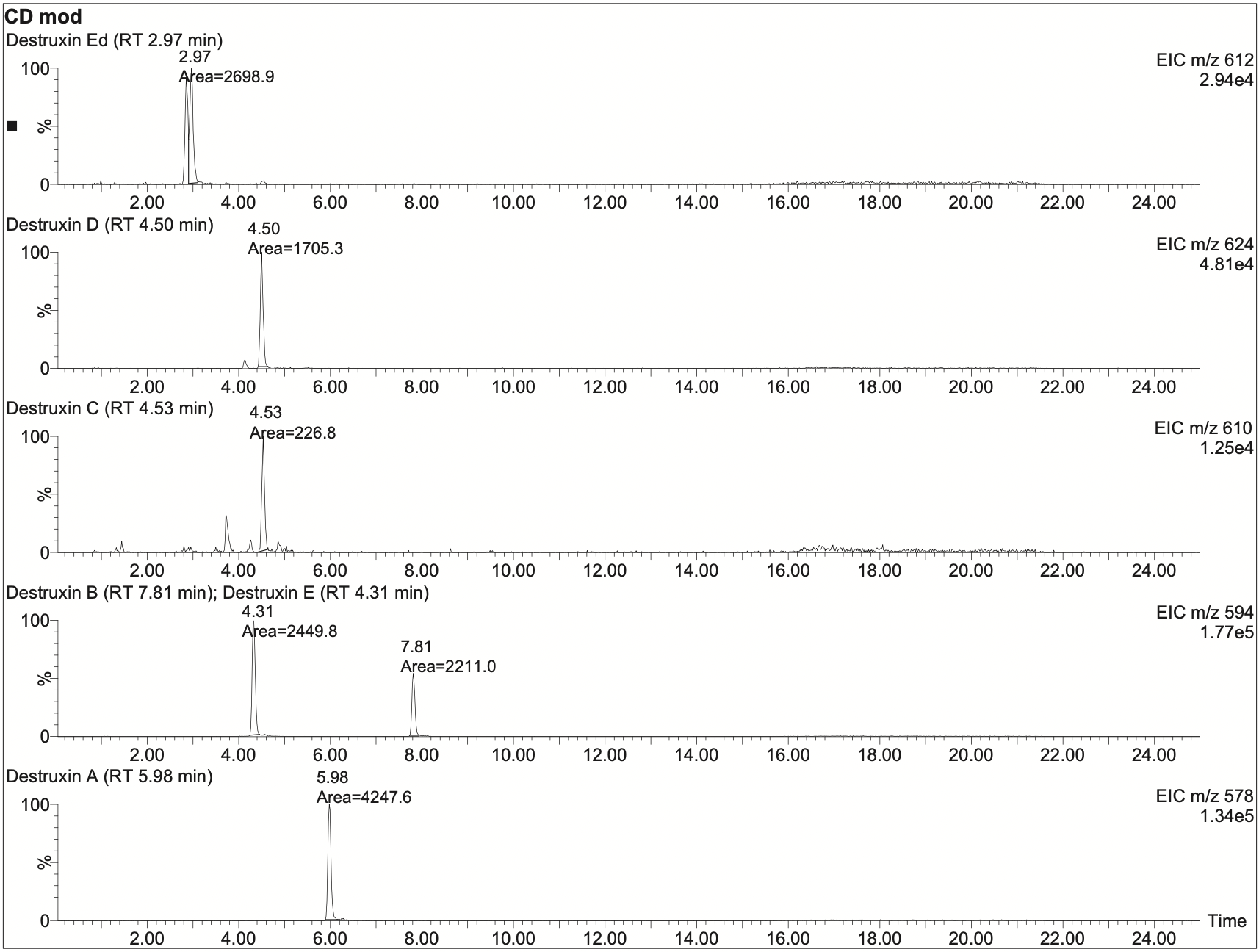
**

**Supplementary Figure S3.** Chromatograms of 5 classes of destruxins in the blastospore culture media: destruxin Ed (m/z= 612), destruxin D (m/z= 624), destruxin C (m/z= 610), destruxin B and E (m/z= 594) and destruxin A (m/z= 578). m/z ratios of the respective destruxins were obtained from Jegorov et al.^9^ and Taibon et al. ^10^. Additionally, structural assignment of destruxins via comparison of MS/MS spectra with reported fragment ions was performed with LC and MS conditions similar to the ones reported in Taibon et al.^10^. See Supplementary Data files for supporting data.

**
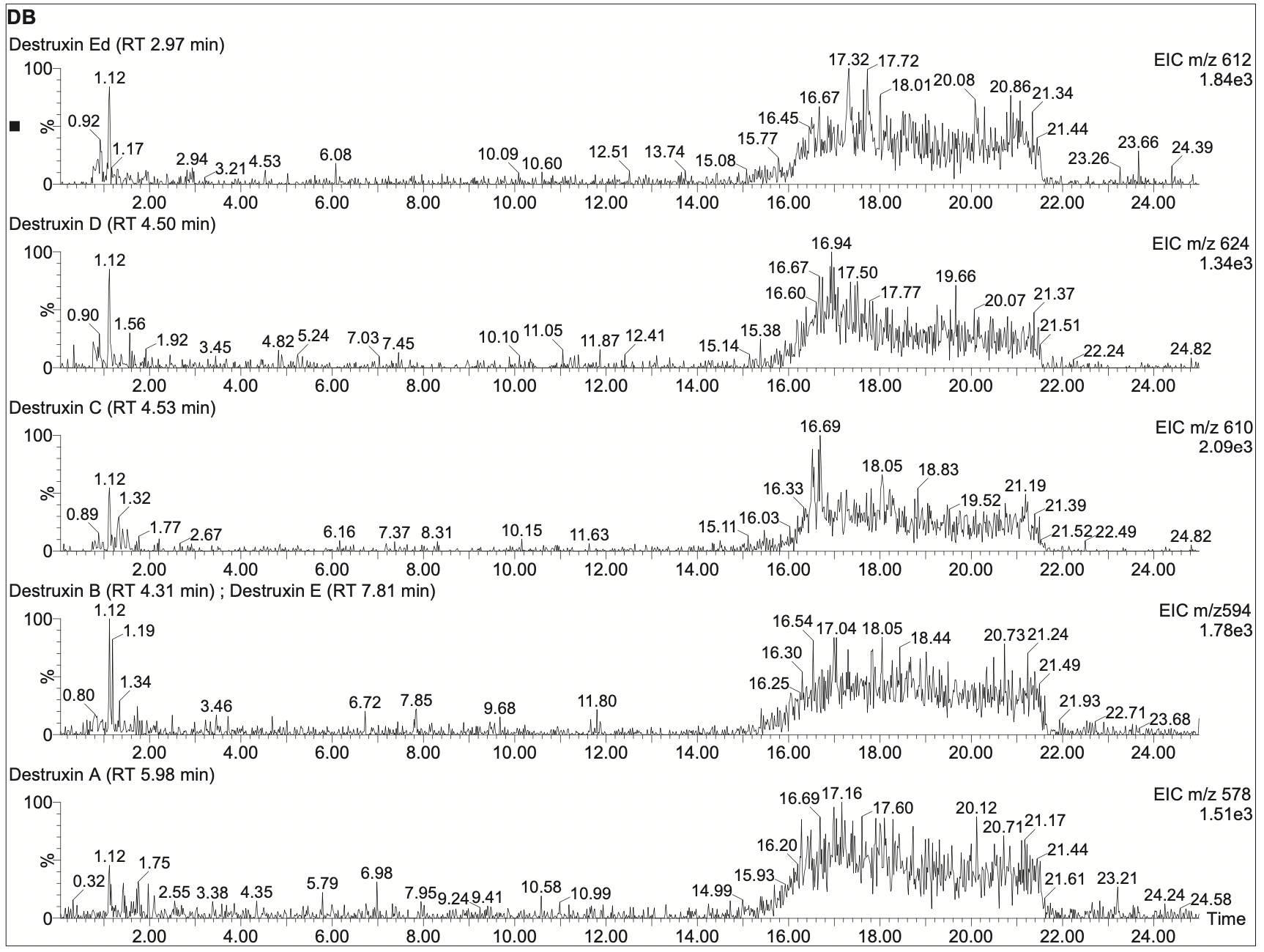
**

**Supplementary Figure S4.** Chromatograms of the filtrate of autoclaved blastospores. None of the 5 classes of destruxins were detectable.


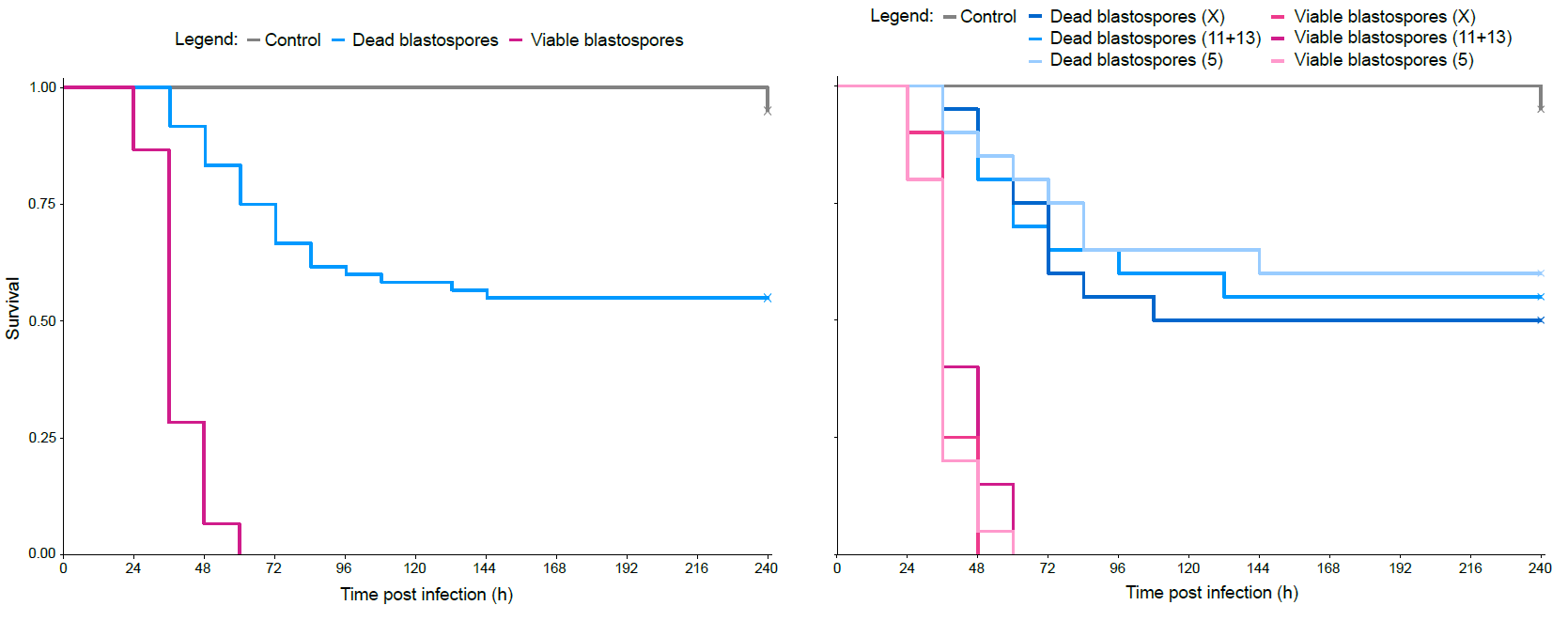


**Supplementary Figure S5.** Survival of individual termites when kept in isolation, by treatment (left) and by treatment and colony (right). The x axis indicates time post injection (in hours) while the y axis depicts survival, calculated as the proportion of surviving termites from each treatment group.

**
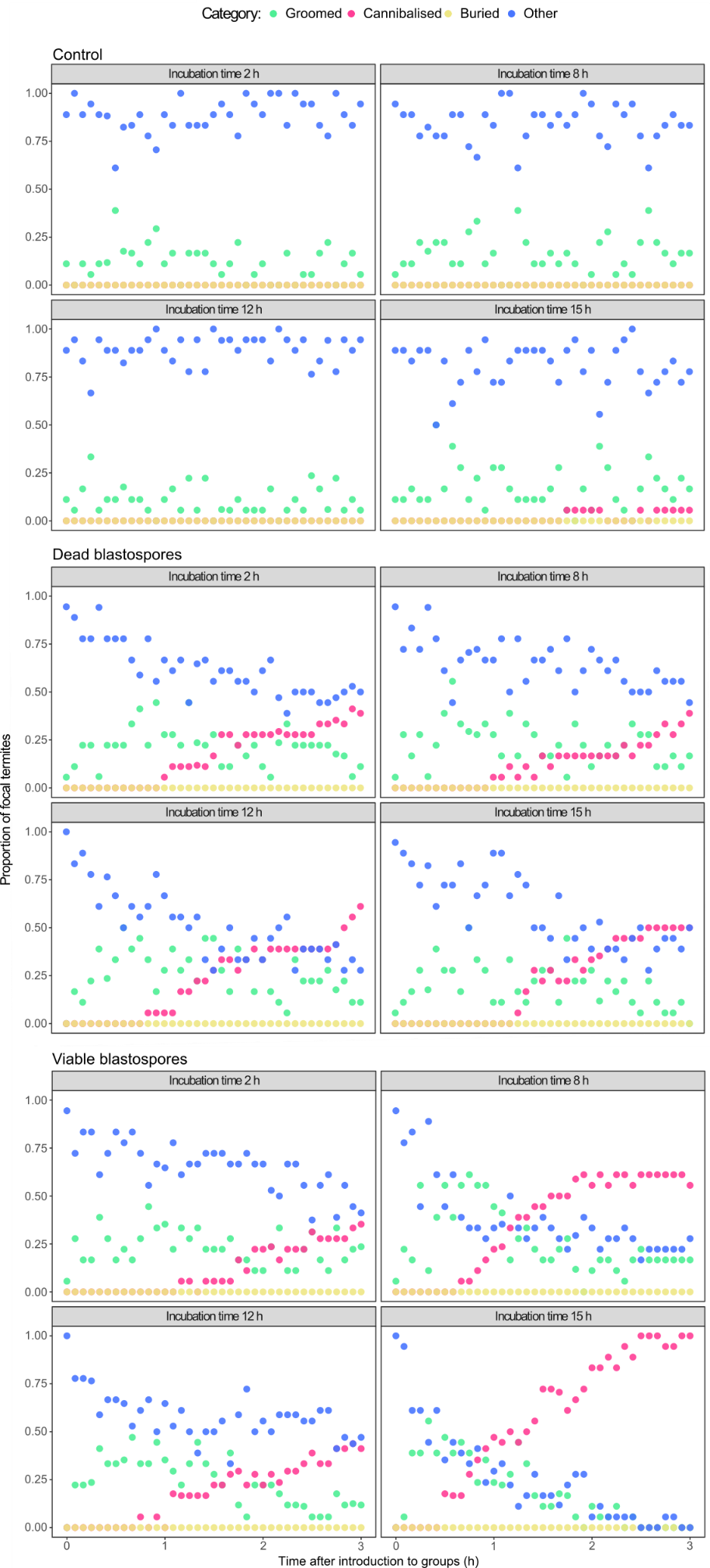
**

**Supplementary Figure S6.** **Blastospore experiment.** The transition of the different behaviours over time during the 3-hours observation period for each of the 3 treatments: control, dead blastospores and viable blastospores. Each point represents the proportion of focal termites that were observed from a given category during a scan at a given timepoint.

**
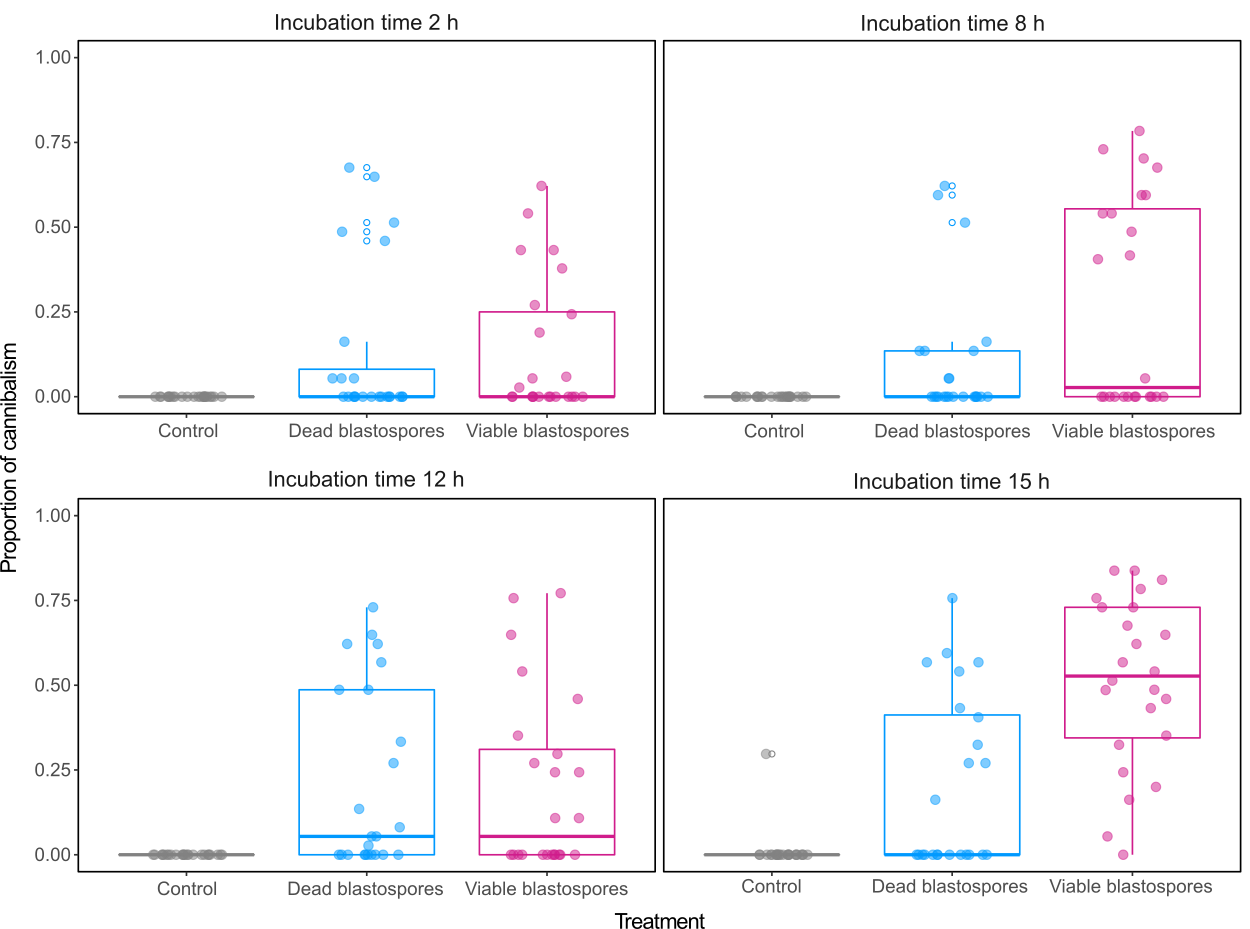
**

**Supplementary Figure S7. Blastospore experiment.** Cannibalism as a proportion of total visible observation states across treatments. Lower and upper hinges correspond to first and third quartiles, the upper whisker extends to the largest value if it is no greater than 1.5 times the inter-quartile rage from the hinge, and the lower whisker extends to the smallest value if it is no smaller than 1.5 times the inter-quartile range from the hinge.

**
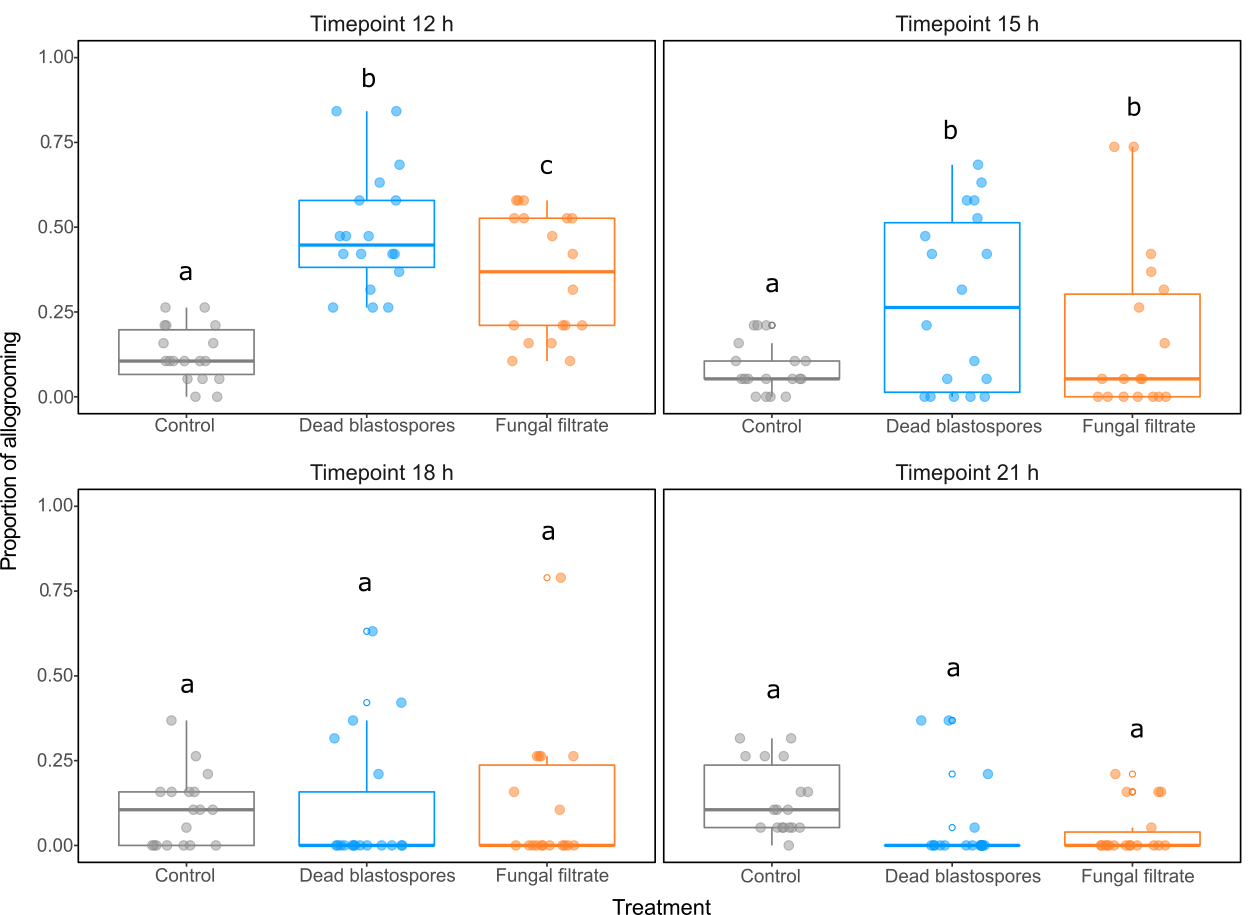
**

**Supplementary Figure S8.** **Blastospore filtrate experiment.** Allogrooming as a proportion of total observation states over time for every treatment (marked with different colors). Treatments marked with different letters were significantly different. Lower and upper hinges indicate first and third quartiles, respectively. The bold middle line represents the median. Whiskers extend to the smallest/largest value if not smaller/greater than 1.5 times the interquartile range (box length).

**
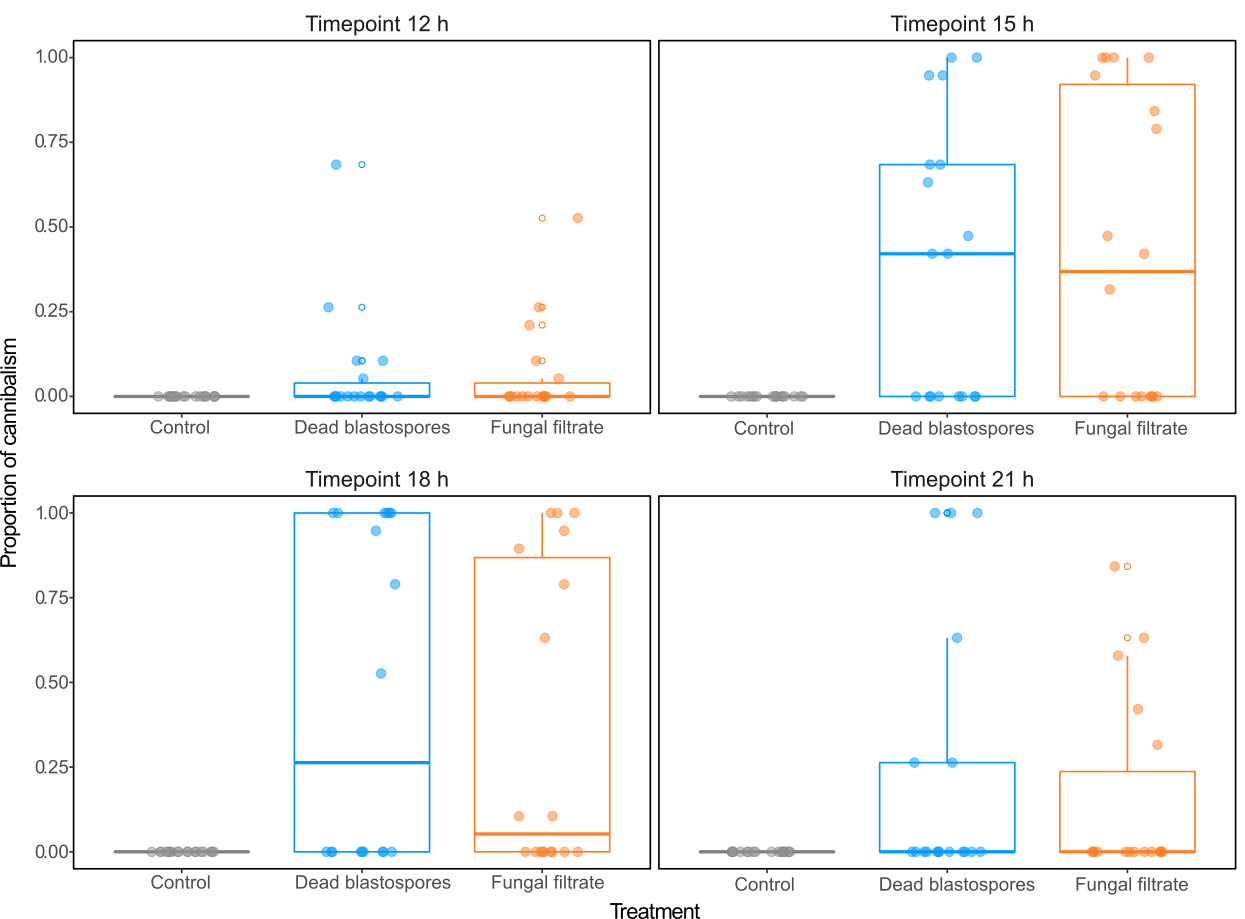
**

**Supplementary Figure S9. Blastospore filtrate experiment.** Cannibalism as a proportion of total visible observation states across treatments. Lower and upper hinges correspond to first and third quartiles, the upper whisker extends to the largest value if it is no greater than 1.5 times the inter-quartile rage from the hinge, and the lower whisker extends to the smallest value if it is no smaller than 1.5 times the inter-quartile range from the hinge.

**
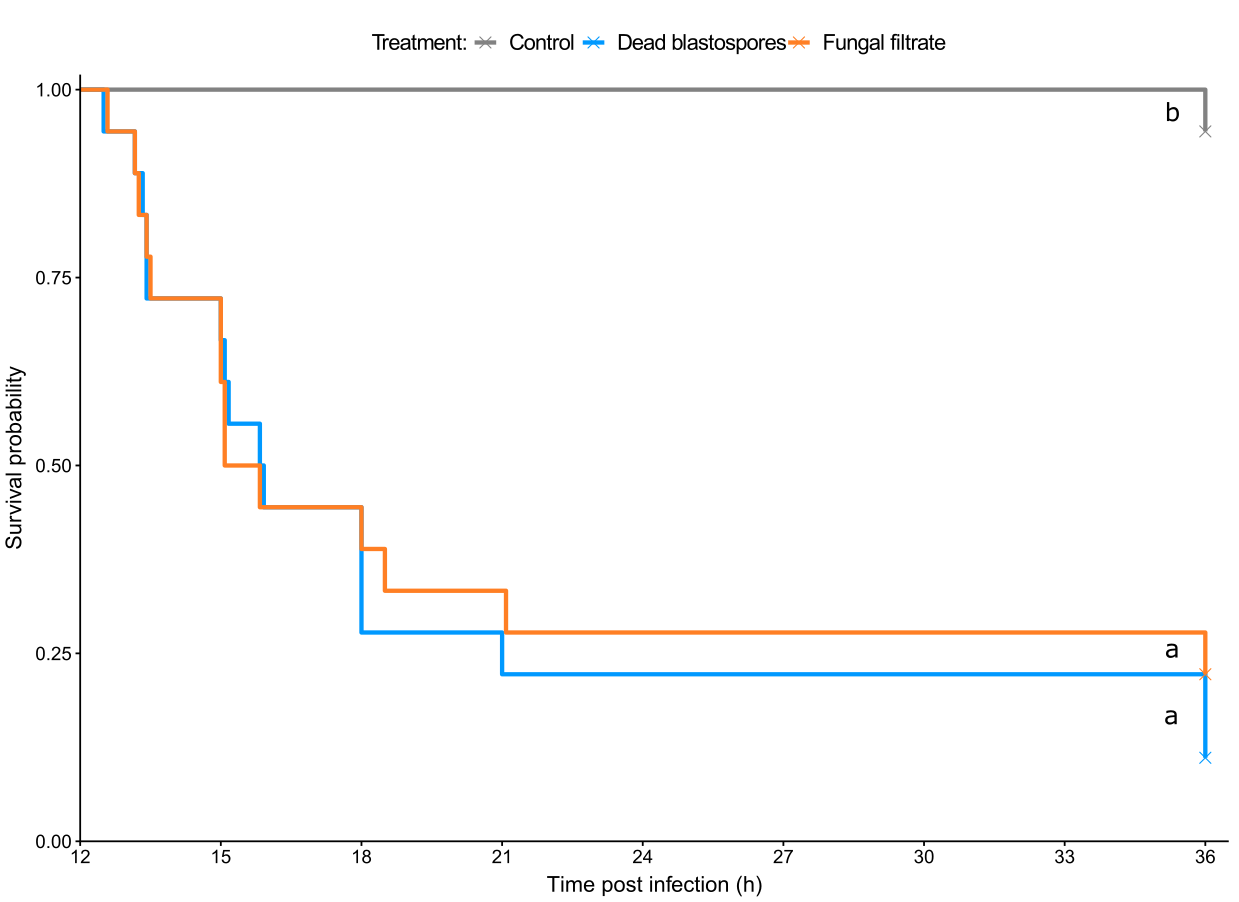
**

**Supplementary Figure S10. Blastospore filtrate experiment.** Survival analysis of the different treatments as represented by different colors. The x axis indicates time post injection (in hours) while the y axis depicts survival, calculated as the proportion of focal termites from every treatment group that had not been cannibalized. Crosses indicate the presence of right-censored data (i.e. focal termites that were not cannibalized during the experiment). A clear difference in survival was detected between the control and the fungal treatments (dead blastospores, fungal filtrate). Treatments marked with the same letter were not significantly different from each other.

Survival assay of different doses of *M. robertsii* blastospores

To select pathogen dose, 120 *Reticulitermes flavipes* workers from colony 5 (20 termites/treatment) were anaesthetized with CO_2_, then injected with 41,4 nL of five different concentrations of viable blastospores (1 x 10^8^, 5 x 10^8^, 8 x 10^8^, 1 x 10^9^ and 3,1 x 10^9^ blastospores/mL) of *Metarhizium robertsii* and Ringer solution (Control) directly into the hemocoel using a Nanoject II (Drummond Scientific Company, USA). Infected termites were kept individually in small Petri dishes (35 mm), each containing a Pall cellulose pad moistened with 1 ml of distilled water and then incubated in darkness for a total of 72 hours at 27 °C. Infected termites were inspected every 12 hours to determine survival.

**
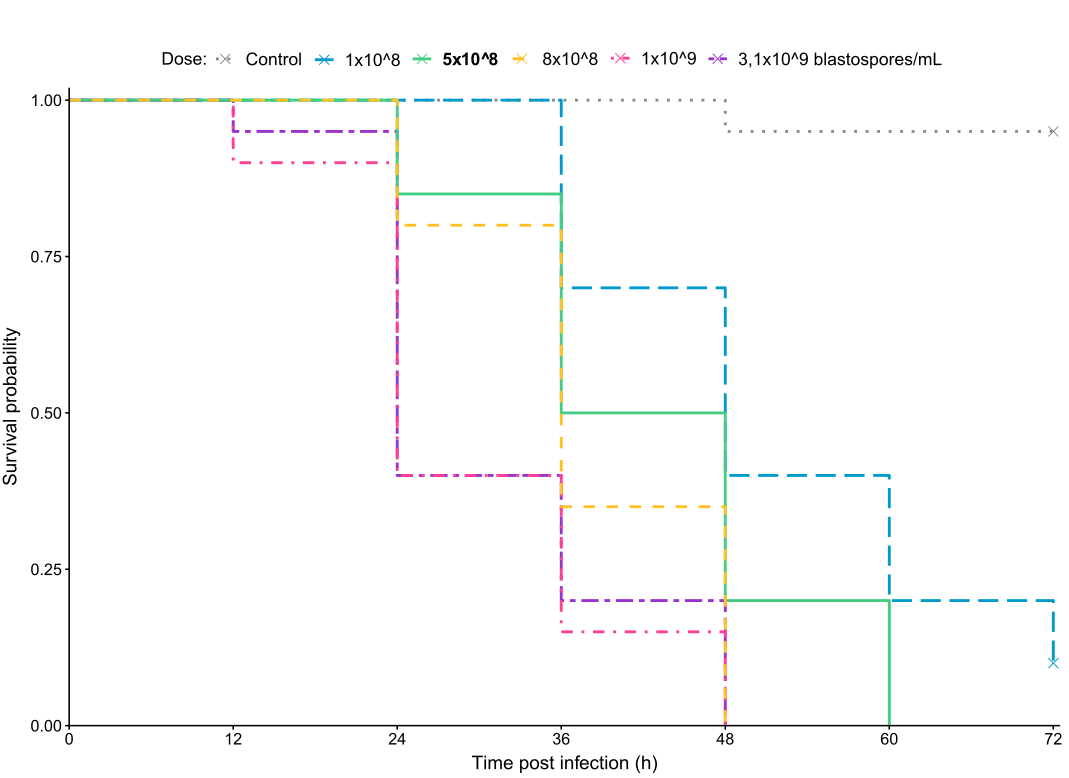
**

**Supplementary Figure S11.** Survival analysis of the different doses of viable blastospores per mL represented by different colors. The x axis indicates time post injection (in hours) while the y axis depicts survival probability, calculated as the proportion of termites from every dose group that were not dead. Crosses indicate the presence of right-censored data (i.e. termites that were not dead during the experiment). In bold (5 x 10^8^ blastospores/mL) represents the selected dose for our experiments.

**Supplementary Table S1. Blastospore experiment.** z and p values from post hoc pairwise comparisons (Tukey tests using a Bonferroni correction) of the proportion of grooming over all observed behaviours. Statistically significant differences are indicated in bold.

| **Incubation time 2 h** | | | |
| --- | --- | --- | --- |
| Treatment | Control (Ringer) | Dead blastospores (Blastospores-) | Viable blastospores  (Blastospores+) |
| Control (Ringer) |  |  |  |
| Dead blastospores (Blastospores-) | **z = 3.293**  ***P* = 0.00297** |  |  |
| Viable blastospores  (Blastospores+) | **z = 4.448**  ***P* = 2.6e-05** | z = 1.247  *P* = 0.63739 |  |
| **Incubation time 8 h** | | | |
| Treatment | Control (Ringer) | Dead blastospores (Blastospores-) | Viable blastospores  (Blastospores+) |
| Control (Ringer) |  |  |  |
| Dead blastospores (Blastospores-) | z = 2.088  *P* = 0.110 |  |  |
| Viable blastospores  (Blastospores+) | z = 2.117  *P* = 0.103 | z = 0.257  *P* = 1.000 |  |
| **Incubation time 12 h** | | | |
| Treatment | Control (Ringer) | Dead blastospores (Blastospores-) | Viable blastospores  (Blastospores+) |
| Control (Ringer) |  |  |  |
| Dead blastospores (Blastospores-) | **z = 4.324**  ***P* = 4.61e-05** |  |  |
| Viable blastospores  (Blastospores+) | **z = 4.515**  ***P* = 1.90e-05** | z = 0.170  *P* = 1 |  |
| **Incubation time 15 h** | | | |
| Treatment | Control (Ringer) | Dead blastospores (Blastospores-) | Viable blastospores  (Blastospores+) |
| Control (Ringer) |  |  |  |
| Dead blastospores (Blastospores-) | z = 1.480  *P* = 0.417 |  |  |
| Viable blastospores  (Blastospores+) | z = 1.359  *P* = 0.522 | z = 0.224  *P* = 1.000 |  |

**Supplementary Table S2.** **Blastospore experiment.** z and p values from post hoc pairwise comparisons (Tukey tests using a Bonferroni correction) of the survival curves, indicating the probability of being cannibalized. Statistically significant differences are indicated in bold.

| **Incubation time 2 h** | | | |
| --- | --- | --- | --- |
| Treatment | Control (Ringer) | Dead blastospores (Blastospores-) | Viable blastospores  (Blastospores+) |
| Control (Ringer) |  |  |  |
| Dead blastospores (Blastospores-) | **z = 3.114**  ***P* =** **0.00553** |  |  |
| Viable blastospores  (Blastospores+) | **z = 3.312**  ***P* = 0.00277** | z = 0.328  *P* = 1.00000 |  |
| **Incubation time 8 h** | | | |
| Treatment | Control (Ringer) | Dead blastospores (Blastospores-) | Viable blastospores  (Blastospores+) |
| Control (Ringer) |  |  |  |
| Dead blastospores (Blastospores-) | **z = 2.397**  ***P* =** **0.04965** |  |  |
| Viable blastospores  (Blastospores+) | **z = 3.226**  ***P* = 0.00377** | z = 1.725  *P* = 0.18504 |  |
| **Incubation time 12 h** | | | |
| Treatment | Control (Ringer) | Dead blastospores (Blastospores-) | Viable blastospores  (Blastospores+) |
| Control (Ringer) |  |  |  |
| Dead blastospores (Blastospores-) | **z = 2.946**  ***P* =** **0.00967** |  |  |
| Viable blastospores  (Blastospores+) | **z = 2.808**  ***P* =** **0.01495** | z = -0.299  *P* = 1.00000 |  |
| **Incubation time 15 h** | | | |
| Treatment | Control (Ringer) | Dead blastospores (Blastospores-) | Viable blastospores  (Blastospores+) |
| Control (Ringer) |  |  |  |
| Dead blastospores (Blastospores-) | **z = 2.684**  ***P* =** **0.021822** |  |  |
| Viable blastospores  (Blastospores+) | **z = 4.822**  ***P* = 4.26e-06** | **z = 4.054**  ***P* =** **0.000151** |  |

**Supplementary Table S3. Blastospore filtrate experiment.** z and p values from post hoc pairwise comparisons (Tukey tests using a Bonferroni correction) of the proportion of grooming over all observed behaviours. Statistically significant differences are indicated in bold.

| **Time point 12 h** | | | |
| --- | --- | --- | --- |
| Treatment | Control (Ringer) | Dead blastospores (Blastospores-) | Fungal filtrate (Filtrate) |
| Control (Ringer) |  |  |  |
| Dead blastospores (Blastospores-) | **z =** **7.636**  ***P* = 6.73e-14** |  |  |
| Fungal filtrate (Filtrate) | **z = 5.349**  ***P* = 2.66e-07** | **z =** **-2.510**  ***P* =** **0.0363** |  |
| **Time point 15 h** | | | |
| Treatment | Control (Ringer) | Dead blastospores (Blastospores-) | Fungal filtrate (Filtrate) |
| Control (Ringer) |  |  |  |
| Dead blastospores (Blastospores-) | **z = 6.626**  ***P* = 1.04e-10** |  |  |
| Fungal filtrate (Filtrate) | **z =** **4.634**  ***P* = 1.08e-05** | z = -2.003  *P* = 0.135 |  |
| **Time point 18 h** | | | |
| **Treatment** | Control (Ringer) | Dead blastospores (Blastospores-) | Fungal filtrate (Filtrate) |
| Control (Ringer) |  |  |  |
| Dead blastospores (Blastospores-) | z = 0.670  *P* = 1.000 |  |  |
| Fungal filtrate (Filtrate) | z = 1.628  *P* = 0.311 | z = 0.765  *P* = 1.000 |  |
| **Time point 21 h** | | | |
| Treatment | Control (Ringer) | Dead blastospores (Blastospores-) | Fungal filtrate (Filtrate) |
| Control (Ringer) |  |  |  |
| Dead blastospores (Blastospores-) | z = -1.967  *P* = 0.1476 |  |  |
| Fungal filtrate (Filtrate) | z = -2.206  *P* = 0.0822 | z = -0.093  *P* = 1.0000 |  |

**Supplementary Table S4. Blastospore filtrate experiment.** z and p values from post hoc pairwise comparisons (Tukey tests using a Bonferroni correction) of the survival curves, indicating the probability of being cannibalized. Statistically significant differences are indicated in bold.

| Treatment | Control (Ringer) | Dead blastospores (Blastospores-) | Fungal filtrate (Filtrate) |
| --- | --- | --- | --- |
| Control (Ringer) |  |  |  |
| Dead blastospores (Blastospores-) | **z =** **3.587**  ***P* = 0.00100** |  |  |
| Fungal filtrate (Filtrate) | **z = 3.339**  ***P* = 0.00253** | z = -0.732  *P* = 1.00000 |  |

Sickness cue observations

To determine appropriate incubation time points, 5 *Reticulitermes flavipes* workers from each colony (11+13, X and 5) were anaesthetized with CO_2_, then injected with 41.4 nL of a suspension of viable blastospores (5 x 10^8^ blastospores/mL) and Ringer solution directly into the hemocoel using a Nanoject II (Drummond Scientific Company, USA). Injection with dead blastospores was carried out in colony 11+13. Injected termites were kept individually in small Petri dishes (35 mm), each containing a Pall cellulose pad moistened with 1 ml of distilled water and then incubated in darkness for a total of 48 hours at 27 °C. Injected termites were observed carefully every 2 hours to determine health status.

| **Incubation time** | **Description: Viable blastospores** | | | | **Dead blastospores** | |
| --- | --- | --- | --- | --- | --- | --- |
|  | Control | Colony 11+13 | Colony X | Colony 5 | Control | Colony 11+13 |
| 0 h | No signs of infection | No signs of infection | No signs of infection | No signs of infection | No signs of infection | No signs of infection |
| 2 h | No signs of infection | No signs of infection | No signs of infection | No signs of infection | No signs of infection | No signs of infection |
| 4 h | No signs of infection | No signs of infection | No signs of infection | No signs of infection | No signs of infection | No signs of infection |
| 6 h | No signs of infection | No signs of infection | No signs of infection | No signs of infection | No signs of infection | No signs of infection |
| 8 h | No signs of infection | No signs of infection | No signs of infection | No signs of infection | No signs of infection | No signs of infection |
| 10 h | No signs of infection | No signs of infection | No signs of infection | No signs of infection | No signs of infection | No signs of infection |
| 12 h | No signs of infection | First signs of weakness | First signs of weakness | First signs of weakness | No signs of infection | No signs of infection |
| 14 h | No signs of infection | ~ | ~ | ~ | No signs of infection | No signs of infection |
| 16 h | No signs of infection | Sick and sluggish | Sick and sluggish | Sick and sluggish | No signs of infection | No signs of infection |
| 18 h | No signs of infection | Very sick / moribund | Sick and sluggish | Sick and sluggish | No signs of infection | No signs of infection |
| 20 h | No signs of infection | Close to death | Sick and sluggish | Very sick / moribund | No signs of infection | No signs of infection |
| 22 h | No signs of infection | Termites start dying | Very sick / moribund | Close to death | No signs of infection | First signs of weakness |
| 24 h | No signs of infection |  | Close to death | Termites start dying | No signs of infection | Look weak |
| 26 h | No signs of infection |  | Termites start dying |  | No signs of infection | Sick and sluggish |
| 36 h | No signs of infection |  |  |  | No signs of infection | Termites start dying |

**Supplementary Table S5.** Description of the symptoms caused by *M. robertsii* when blastospores are injected directly into the termite hemocoel. Different colors (light blue, pink, green and yellow) represent varying signs of disease observed after injection of blastospores. “Signs of weakness”; “Very sick/moribund”; “close to death” refer to the following behaviours, respectively: slower walking; intermittent/difficult walking; antennal movements only or leg movements if lying dorsally.

References

1. Perdereau, E. et al. Invasion Dynamics of A Termite, Reticulitermes flavipes, at Different Spatial Scales in France. Insects 10, 30 (2019).
2. Vargo, E. L. & Husseneder, C. Biology of subterranean termites: insights from molecular studies of Reticulitermes and Coptotermes. Annual Review of Entomology 54, 379-403 (2009).80 Becker, G. Rearing of termites and testing methods used in the laboratory. In Biology of Termites (Volume 1, eds K. Krishna and F. M. Weesner), pp. 351–385. Academic Press, New York. (1969).
3. Dawes-Gromadzki, T. Z. Sampling subterranean termite species diversity and activity in tropical savannas: an assessment of different bait choices. Ecological Entomology 28, 397-404 (2003).
4. Denier, D. & Bulmer, M.S. Variation in subterranean termite susceptibility to fatal infections by local *Metarhizium* soil isolates. *Insectes Sociaux* 62, 219-226 (2015).
5. Adamek, L. SUBMERSE CULTIVATION OF THE FUNGUS *METARRHIZIUM ANISOPLIAE* (METSCH.). *Folia Microbiologica* 10, 255-257 (1965).
6. Bernardo, C. C. et al. Conidia and blastospores of *Metarhizium* spp. and *Beauveria bassiana* s.l.: Their development during the infection process and virulence against the tick Rhipicephalus microplus. *Ticks and Tick-borne Diseases* 9, 1334-1342 (2018).
7. Aguero, C. M., Eyer, P.-A. & Vargo, E. L. Increased genetic diversity from colony merging in termites does not improve survival against a fungal pathogen. *Scientific Reports* 10, 4212 (2020).
8. Davis, H. E., Meconcelli, S., Radek, R. & McMahon, D. P. Termites shape their collective behavioural response based on stage of infection. *Scientific reports* 8, 1-10 (2018).
9. Jegorov 1998: A. Jegorov, V. Havlíček and P. Sedmera: Rapid screening of destruxins by liquid chromatography/mass spectrometry, J. Mass Spectrom. 33(3), 274-280 (1998) DOI: 10.1002/(SICI)1096-9888(199803)33:3<274::AID-JMS630>3.0.CO;2-R
10. Taibon 2014: J. Taibon, S. Sturm, C. Seger, M. Parth, H. Strasser and H. Stuppner: Development of a fast and selective UHPLC-DAD-QTOF-MS/MS method for the qualitative and quantitative assessment of destruxin profiles, Anal. Bioanal. Chem. 406(29), 7623-7632 (2014) DOI: 10.1007/s00216-014-8203-z
