# Supplementary material for: Defensive behavior is linked to altered surface chemistry following infection in a termite society
Source: Sci Rep. 2023 Nov 23;13:20606. doi: 10.1038/s41598-023-42947-9 (PMC10667546; doi:10.1038/s41598-023-42947-9)
Supplement: Supplementary file 2 — Supplementary Information 2. [file 41598_2023_42947_MOESM2_ESM.zip › Data/Data/Destruxin Czepek Dox modified media.zip!/Czepek Dox modified media/[CDmod]_MSMS_578.3 at 6.06min.pdf]

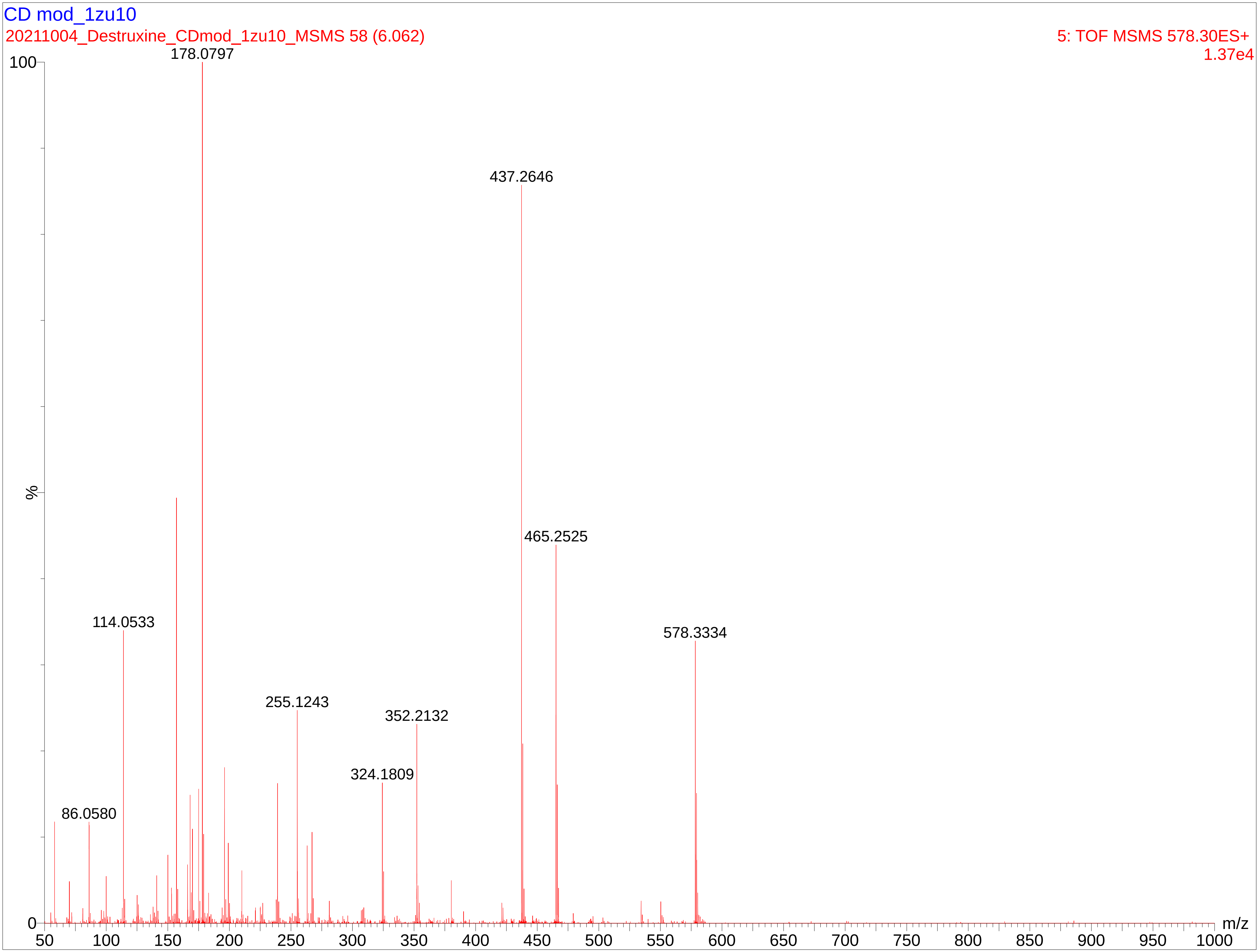
CD mod_1zu10
20211004_Destruxine_CDmod_1zu10_MSMS 58 (6.062)
5: TOF MSMS 578.30ES+
1.37e4
178.0797
437.2646
465.2525
114.0533
578.3334
255.1243
352.2132
324.1809
86.0580
100
%
0
m/z
50
100
150
200
250
300
350
400
450
500
550
600
650
700
750
800
850
900
950
1000
