# Supplementary material for: Defensive behavior is linked to altered surface chemistry following infection in a termite society
Source: Sci Rep. 2023 Nov 23;13:20606. doi: 10.1038/s41598-023-42947-9 (PMC10667546; doi:10.1038/s41598-023-42947-9)
Supplement: Supplementary file 2 — Supplementary Information 2. [file 41598_2023_42947_MOESM2_ESM.zip › Data/Data/Destruxin Czepek Dox modified media.zip!/Czepek Dox modified media/[CDmod]_MSMS_594.3 at 7.80min.pdf]

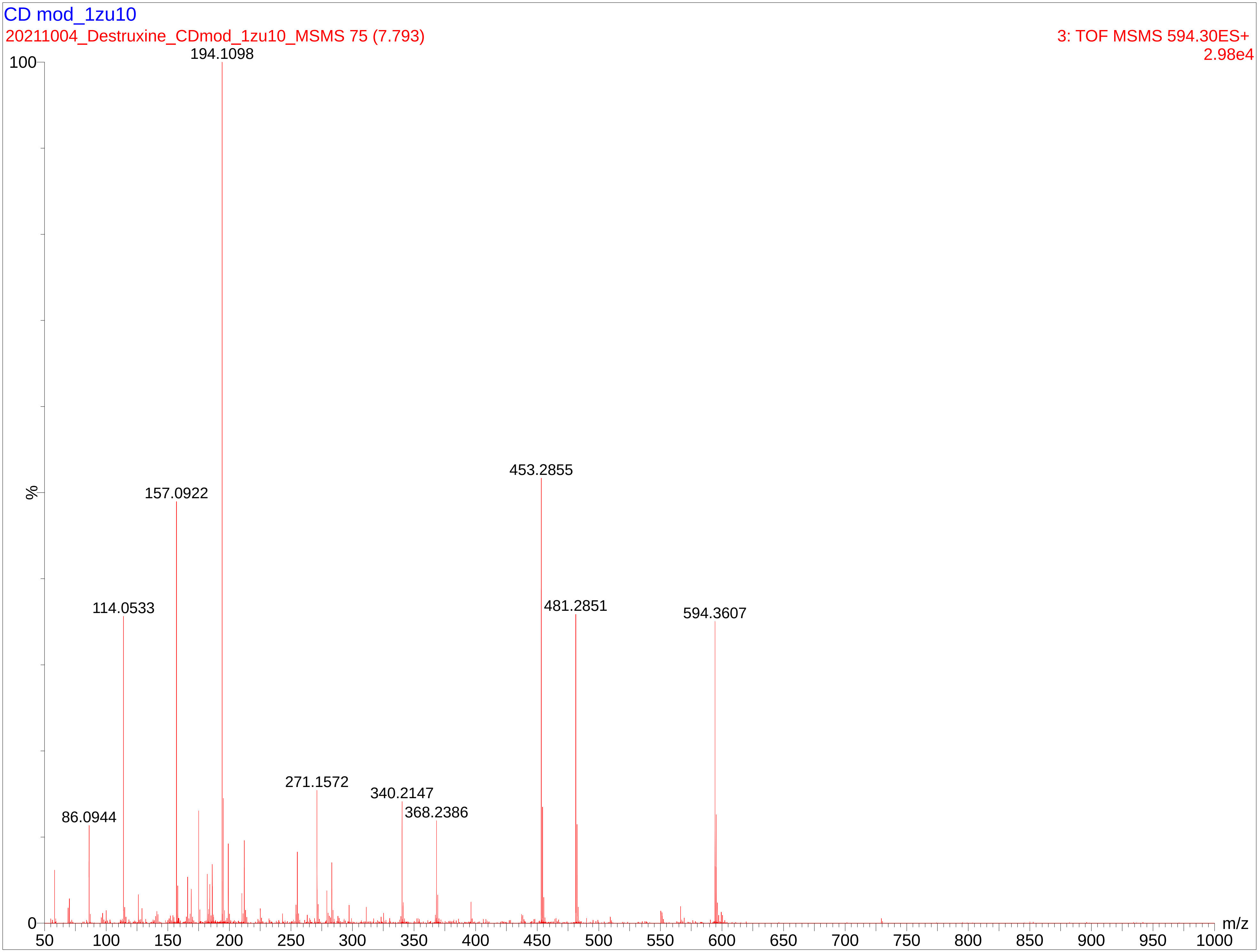
CD mod_1zu10
20211004_Destruxine_CDmod_1zu10_MSMS 75 (7.793)
3: TOF MSMS 594.30ES+
2.98e4
100
%
0
194.1098
157.0922
114.0533
86.0944
271.1572
340.2147
368.2386
453.2855
481.2851
594.3607
50
100
150
200
250
300
350
400
450
500
550
600
650
700
750
800
850
900
950
1000
m/z
