# Supplementary material for: Defensive behavior is linked to altered surface chemistry following infection in a termite society
Source: Sci Rep. 2023 Nov 23;13:20606. doi: 10.1038/s41598-023-42947-9 (PMC10667546; doi:10.1038/s41598-023-42947-9)
Supplement: Supplementary file 2 — Supplementary Information 2. [file 41598_2023_42947_MOESM2_ESM.zip › Data/Data/Destruxin Czepek Dox modified media.zip!/Czepek Dox modified media/[CDmod]_MSMS_610.3 at 3.82min.pdf]

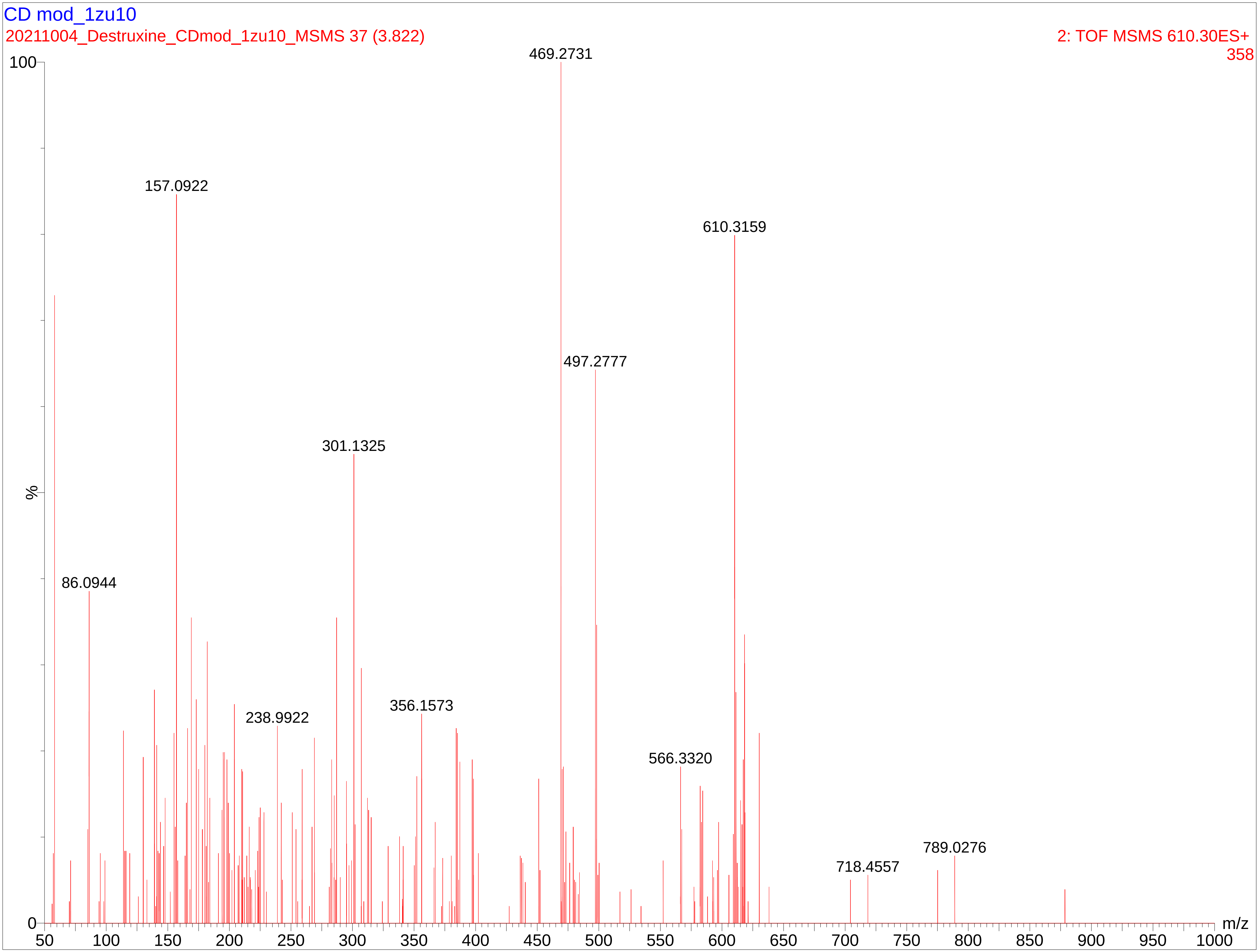

CD mod_1zu10
20211004_Destruxine_CDmod_1zu10_MSMS 37 (3.822)
2: TOF MSMS 610.30ES+
358
469.2731
157.0922
610.3159
497.2777
301.1325
86.0944
356.1573
238.9922
566.3320
789.0276
718.4557
100
%
0
m/z
50
100
150
200
250
300
350
400
450
500
550
600
650
700
750
800
850
900
950
1000
