# Supplementary material for: Defensive behavior is linked to altered surface chemistry following infection in a termite society
Source: Sci Rep. 2023 Nov 23;13:20606. doi: 10.1038/s41598-023-42947-9 (PMC10667546; doi:10.1038/s41598-023-42947-9)
Supplement: Supplementary file 2 — Supplementary Information 2. [file 41598_2023_42947_MOESM2_ESM.zip › Data/Data/Destruxin Czepek Dox modified media.zip!/Czepek Dox modified media/[CDmod]_MSMS_612.3 at 2.95min.pdf]

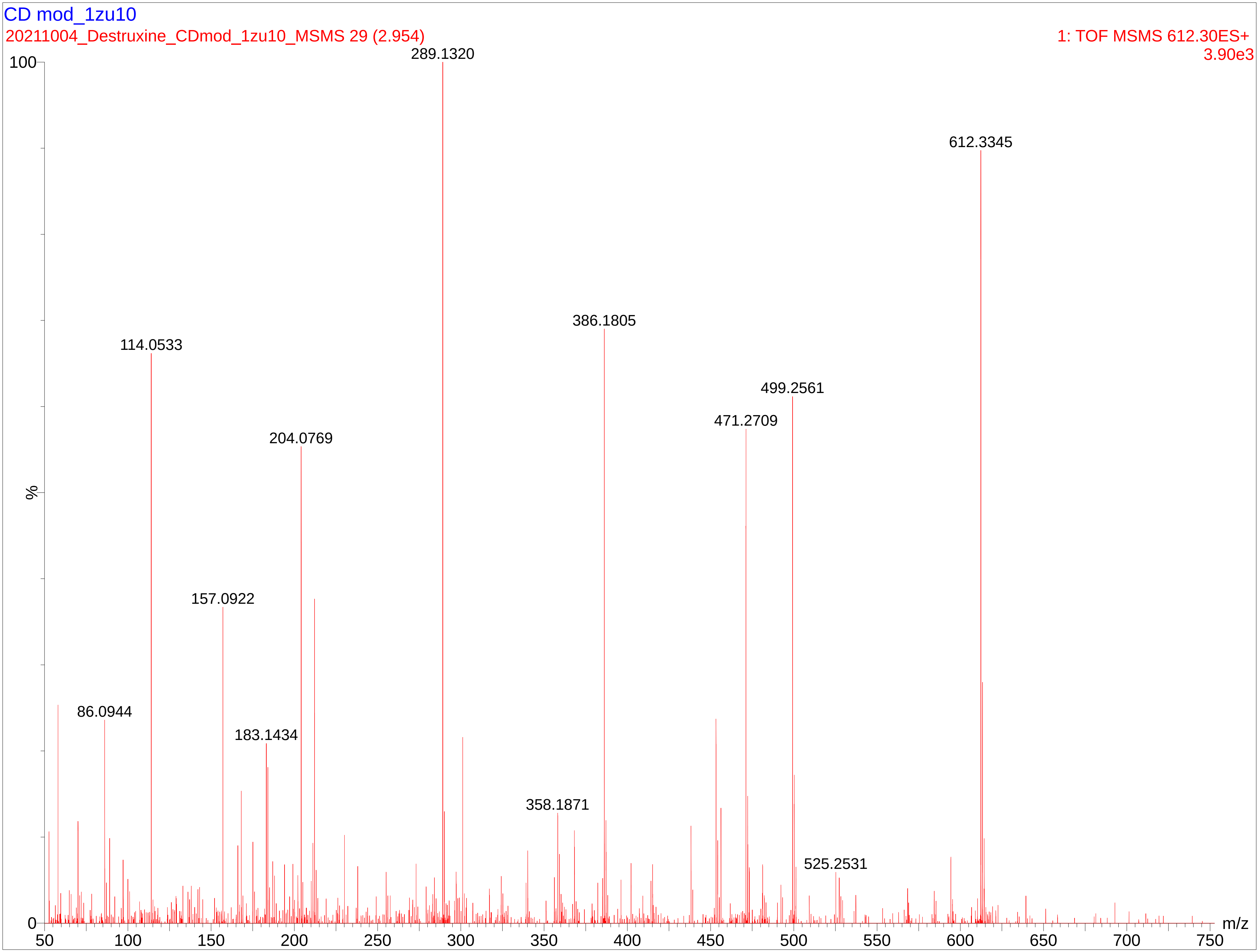
CD mod_1zu10
20211004_Destruxine_CDmod_1zu10_MSMS 29 (2.954)
1: TOF MSMS 612.30ES+
3.90e3
289.1320
612.3345
386.1805
114.0533
499.2561
471.2709
204.0769
157.0922
86.0944
183.1434
358.1871
525.2531
100
%
0
m/z
50
100
150
200
250
300
350
400
450
500
550
600
650
700
750
