# Supplementary material for: Defensive behavior is linked to altered surface chemistry following infection in a termite society
Source: Sci Rep. 2023 Nov 23;13:20606. doi: 10.1038/s41598-023-42947-9 (PMC10667546; doi:10.1038/s41598-023-42947-9)
Supplement: Supplementary file 2 — Supplementary Information 2. [file 41598_2023_42947_MOESM2_ESM.zip › Data/Data/Destruxin Czepek Dox modified media.zip!/Czepek Dox modified media/[CDmod]_MSMS_624.3 at 4.59min.pdf]

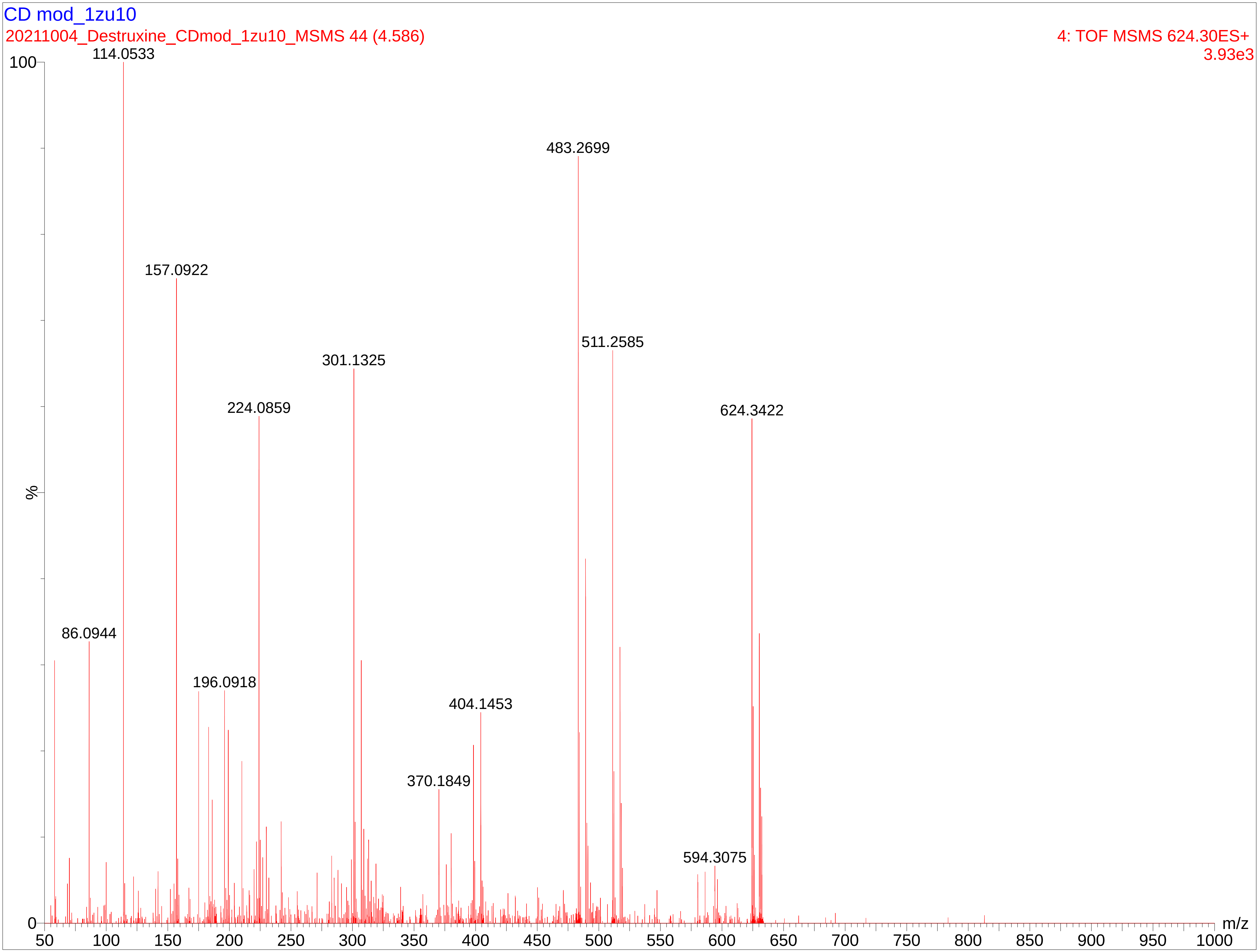

CD mod_1zu10
20211004_Destruxine_CDmod_1zu10_MSMS 44 (4.586)
4: TOF MSMS 624.30ES+
3.93e3
114.0533
483.2699
157.0922
511.2585
301.1325
224.0859
624.3422
86.0944
196.0918
404.1453
370.1849
594.3075
100
%
0
50
100
150
200
250
300
350
400
450
500
550
600
650
700
750
800
850
900
950
1000
m/z
