# Supplementary material for: Defensive behavior is linked to altered surface chemistry following infection in a termite society
Source: Sci Rep. 2023 Nov 23;13:20606. doi: 10.1038/s41598-023-42947-9 (PMC10667546; doi:10.1038/s41598-023-42947-9)
Supplement: Supplementary file 2 — Supplementary Information 2. [file 41598_2023_42947_MOESM2_ESM.zip › Data/Data/Destruxin Czepek Dox modified media.zip!/Czepek Dox modified media/[CDmod]_MS_all.pdf]

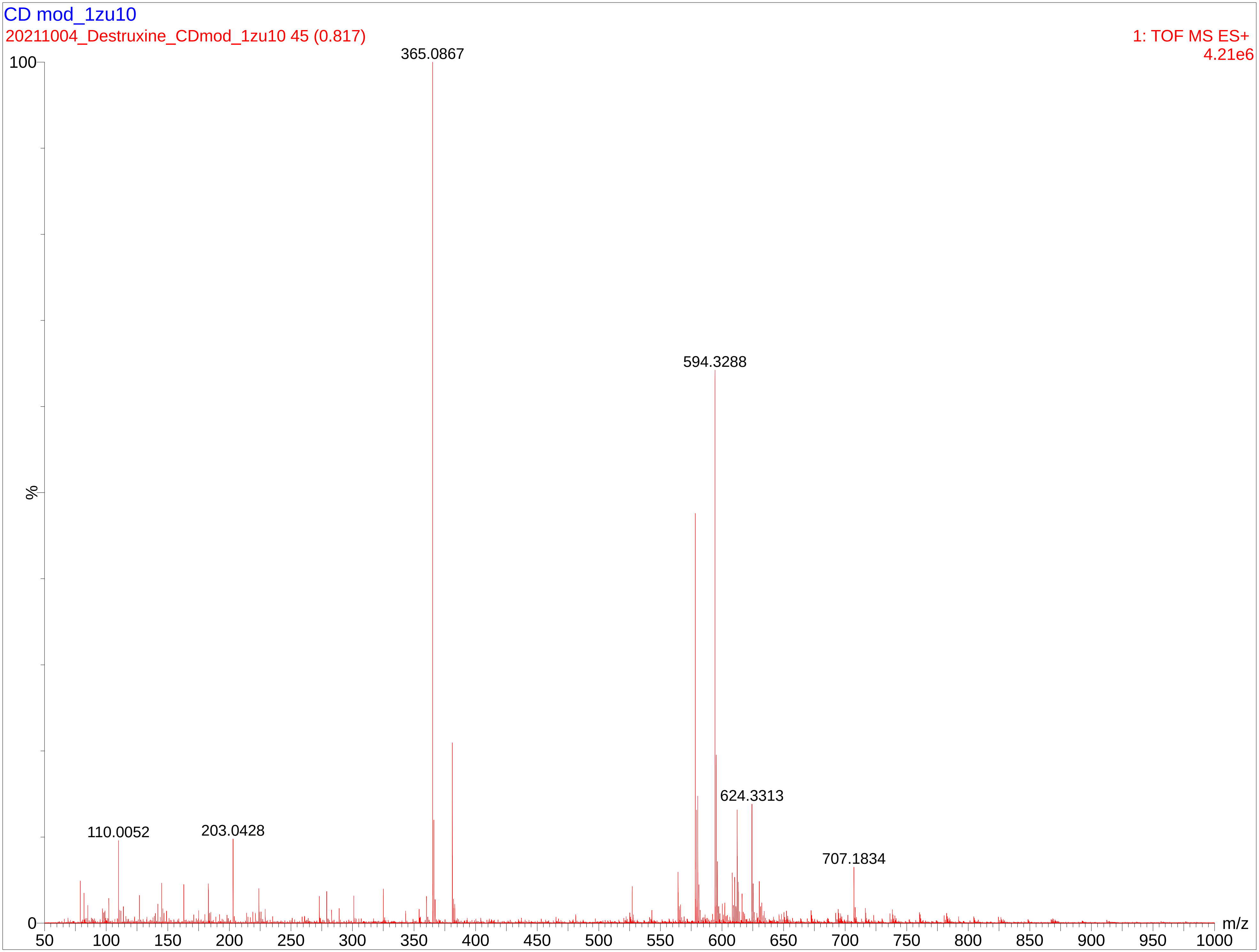
CD mod_1zu10
20211004_Destruxine_CDmod_1zu10 45 (0.817)
1: TOF MS ES+
4.21e6
365.0867
594.3288
624.3313
707.1834
110.0052
203.0428
100
%
0
50
100
150
200
250
300
350
400
450
500
550
600
650
700
750
800
850
900
950
1000
m/z
