# Supplementary material for: Defensive behavior is linked to altered surface chemistry following infection in a termite society
Source: Sci Rep. 2023 Nov 23;13:20606. doi: 10.1038/s41598-023-42947-9 (PMC10667546; doi:10.1038/s41598-023-42947-9)
Supplement: Supplementary file 2 — Supplementary Information 2. [file 41598_2023_42947_MOESM2_ESM.zip › Data/Data/Destruxin Czepek Dox modified media.zip!/Czepek Dox modified media/[CDmod]_MS_all_zoom.pdf]

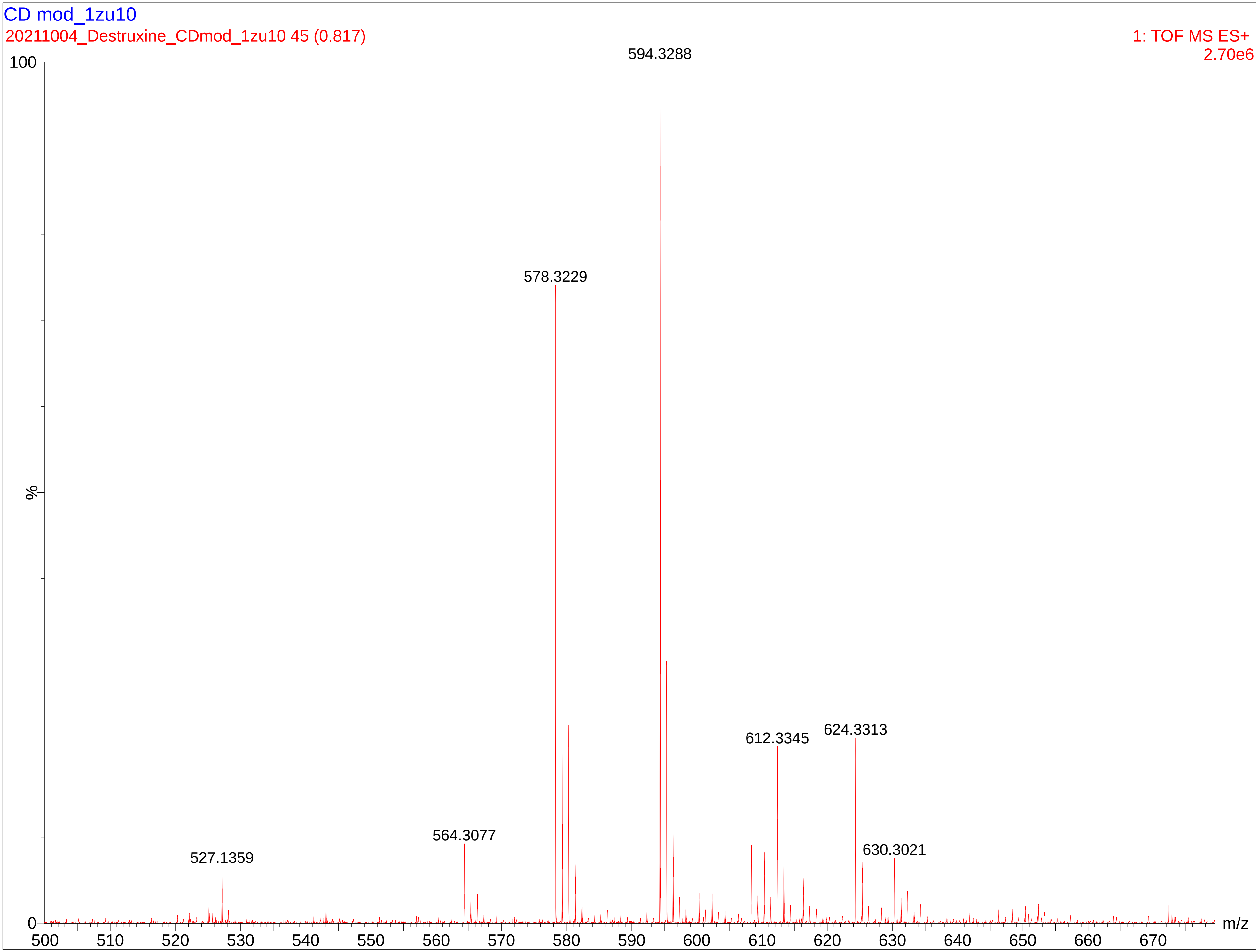
CD mod_1zu10
20211004_Destruxine_CDmod_1zu10 45 (0.817)
1: TOF MS ES+
2.70e6
594.3288
578.3229
612.3345
624.3313
564.3077
630.3021
527.1359
100
%
0
500
510
520
530
540
550
560
570
580
590
600
610
620
630
640
650
660
670
m/z
