# Supplementary material for: Defensive behavior is linked to altered surface chemistry following infection in a termite society
Source: Sci Rep. 2023 Nov 23;13:20606. doi: 10.1038/s41598-023-42947-9 (PMC10667546; doi:10.1038/s41598-023-42947-9)
Supplement: Supplementary file 2 — Supplementary Information 2. [file 41598_2023_42947_MOESM2_ESM.zip › Data/Data/Destruxin Czepek Dox modified media.zip!/Czepek Dox modified media/[CDmod]_MS_retention time.pdf]

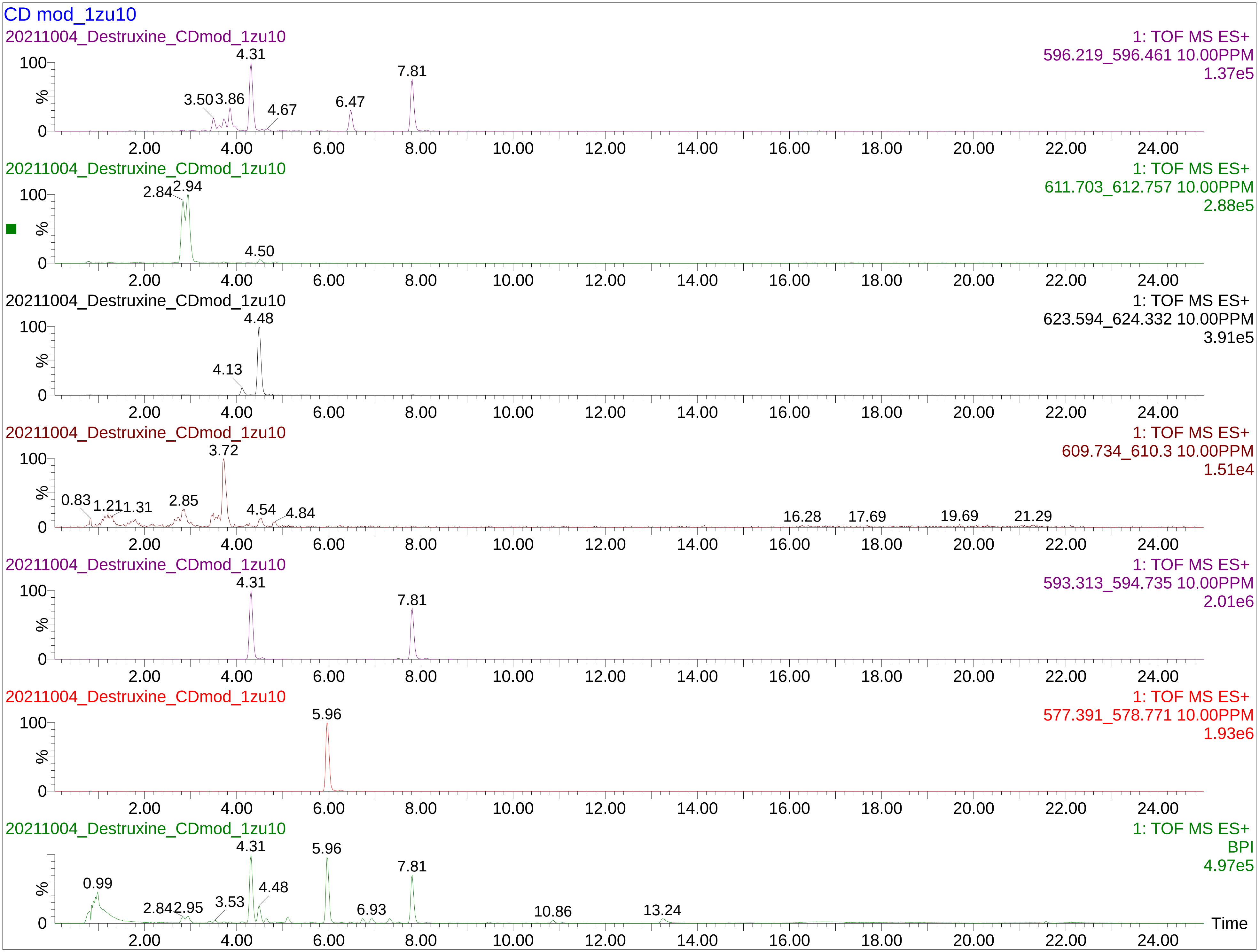
CD mod_1zu10
20211004_Destruxine_CDmod_1zu10
1: TOF MS ES+
596.219_596.461 10.00PPM
1.37e5
4.31
7.81
3.50
3.86
4.67
6.47
20211004_Destruxine_CDmod_1zu10
1: TOF MS ES+
611.703_612.757 10.00PPM
2.88e5
2.84
2.94
4.50
20211004_Destruxine_CDmod_1zu10
1: TOF MS ES+
623.594_624.332 10.00PPM
3.91e5
4.48
4.13
20211004_Destruxine_CDmod_1zu10
1: TOF MS ES+
609.734_610.3 10.00PPM
1.51e4
3.72
0.83
1.21
1.31
2.85
4.54
4.84
16.28
17.69
19.69
21.29
20211004_Destruxine_CDmod_1zu10
1: TOF MS ES+
593.313_594.735 10.00PPM
2.01e6
4.31
7.81
20211004_Destruxine_CDmod_1zu10
1: TOF MS ES+
577.391_578.771 10.00PPM
1.93e6
5.96
20211004_Destruxine_CDmod_1zu10
1: TOF MS ES+
BPI
4.97e5
4.31
5.96
7.81
0.99
2.84
2.95
3.53
4.48
6.93
10.86
13.24
%
100
0
2.00
4.00
6.00
8.00
10.00
12.00
14.00
16.00
18.00
20.00
22.00
24.00
Time
