# Supplementary material for: Defensive behavior is linked to altered surface chemistry following infection in a termite society
Source: Sci Rep. 2023 Nov 23;13:20606. doi: 10.1038/s41598-023-42947-9 (PMC10667546; doi:10.1038/s41598-023-42947-9)
Supplement: Supplementary file 2 — Supplementary Information 2. [file 41598_2023_42947_MOESM2_ESM.zip › Data/Data/Destruxin Czepek Dox modified media.zip!/Czepek Dox modified media/[CDmod]_TIC.pdf]

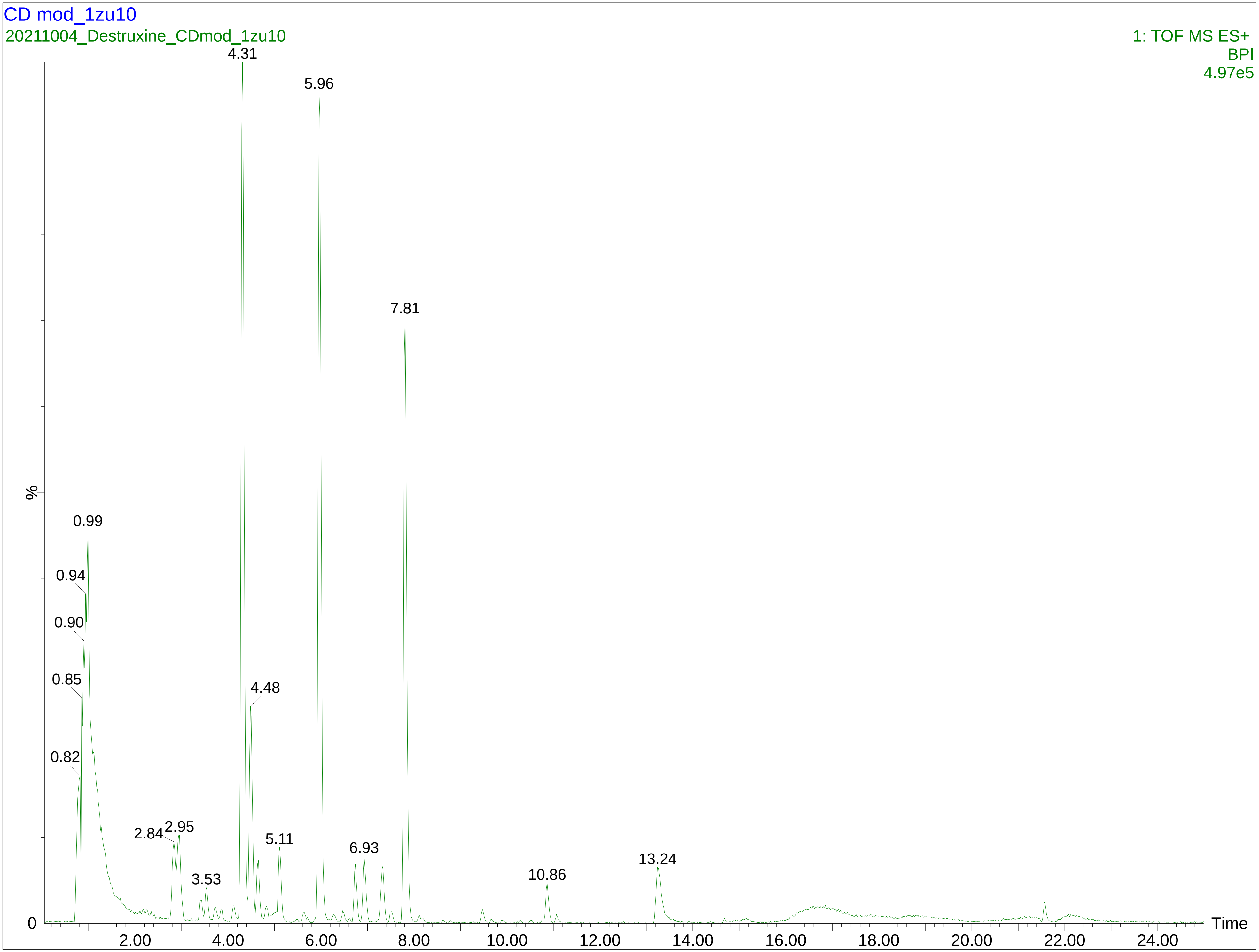

CD mod_1zu10
20211004_Destruxine_CDmod_1zu10
1: TOF MS ES+
BPI
4.97e5
%
0
4.31
5.96
7.81
0.99
0.94
0.90
0.85
0.82
4.48
2.84
2.95
5.11
6.93
3.53
10.86
13.24
2.00
4.00
6.00
8.00
10.00
12.00
14.00
16.00
18.00
20.00
22.00
24.00
Time
